# Renewable production of high density jet fuel precursor sesquiterpenes from *Escherichia coli*

**DOI:** 10.1186/s13068-018-1272-z

**Published:** 2018-10-20

**Authors:** Chun-Li Liu, Tian Tian, Jorge Alonso-Gutierrez, Brett Garabedian, Shuai Wang, Edward E. K. Baidoo, Veronica Benites, Yan Chen, Christopher J. Petzold, Paul D. Adams, Jay D. Keasling, Tianwei Tan, Taek Soon Lee

**Affiliations:** 1 College of Life Science and Technology, Beijing University of Chemical Technology, Beijing, 100029 People’s Republic of China; 2 Joint BioEnergy Institute, 5885 Hollis Street, 4th Floor, Emeryville, CA 94608 USA; 3 Biological Systems & Engineering Division, Lawrence Berkeley National Laboratory, Berkeley, CA 94720 USA; 4 Department of Bioengineering, University of California, Berkeley, CA 94720 USA; 5 Department of Chemical and Biomolecular Engineering, University of California, Berkeley, CA 94720 USA; 6 The Novo Nordisk Foundation Center for Biosustainability, Technical University of Denmark, Lyngby, Denmark; 7 Center for Synthetic Biochemistry, Shenzhen Institutes for Advanced Technologies, Shenzhen, China

**Keywords:** Sesquiterpene, Jet fuel, Epi-isozizaene, Pentalenene, α-Isocomene, FPP-responsive promoter engineering

## Abstract

**Background:**

Aviation fuels are an important target of biofuels research due to their high market demand and competitive price. Isoprenoids have been demonstrated as good feedstocks for advanced renewable jet fuels with high energy density, high heat of combustion, and excellent cold-weather performance. In particular, sesquiterpene compounds (C_15_), such as farnesene and bisabolene, have been identified as promising jet fuel candidates.

**Results:**

In this study, we explored three sesquiterpenes—epi-isozizaene, pentalenene and α-isocomene—as novel jet fuel precursors. We performed a computational analysis to calculate the energy of combustion of these sesquiterpenes and found that their specific energies are comparable to commercial jet fuel A-1. Through heterologous MVA pathway expression and promoter engineering, we produced 727.9 mg/L epi-isozizaene, 780.3 mg/L pentalenene and 77.5 mg/L α-isocomene in *Escherichia coli* and 344 mg/L pentalenene in *Saccharomyces cerevisiae*. We also introduced a dynamic autoinduction system using previously identified FPP-responsive promoters for inducer-free production and managed to achieve comparable amounts of each compound.

**Conclusion:**

We produced tricyclic sesquiterpenes epi-isozizaene, pentalenene and α-isocomene, promising jet fuel feedstocks at high production titers, providing novel, sustainable alternatives to petroleum-based jet fuels.

**Electronic supplementary material:**

The online version of this article (10.1186/s13068-018-1272-z) contains supplementary material, which is available to authorized users.

## Background

Several microbial platforms have been developed for advanced biofuel production [1–3]. The advanced biofuels, derived from higher alcohols, alkanes/alkenes, fatty acid esters and isoprenoids, are sustainable energy alternatives, with favorable properties and great market potential [3–5]. Among these biofuel candidates, C_10_ and C_15_ terpenes (monoterpenes and sesquiterpenes) have been increasingly used as jet fuel alternatives for commercial aviation and military purpose because of their structures, suitable carbon numbers and reactive olefin functionality, which collectively impart low freezing points and high energy densities [6–8]. For example, monoterpenes or monoterpenoids, such as pinenes [9], linalool [10] and limonene [11] have been used as biosynthetic precursors for aviation and missile fuels such as JP-10, RJ-4 and Jet A-1. Additionally, the hydrogenated sesquiterpene, farnesane, has been commercialized as a blend stock for jet fuel AMJ-700 [12]. Recently, there has been significant interest in multicyclic sesquiterpenes as next-generation jet fuel substitutes due to their high energy density and comparable cetane numbers [13]. For example, three sesquiterpenes, thujopsene, α-cedrene, and β-cedrene, were hydrogenated to generate a fuel blend with 12% higher volumetric net heat of combustion than conventional jet fuel [13].

In the interest of expanding the scope of multicyclic hydrocarbons as jet fuel alternatives, we sought to produce three novel tricyclic sesquiterpenes: epi-isozizaene, pentalenene and α-isocomene. These sesquiterpenes were primarily identified as antibiotic metabolites [14]. Epi-isozizaene and pentalenene are produced by several *Streptomyces* species [15–18], while α-isocomene is mainly isolated from plants such as *Isocoma wright*, *Berkheya radulu* and *Matricaria recutita* [19–21]. Their production and recovery efficiency from the natural producers, however, are very low when considering the quantity and the purity. In this study, we propose that these multicyclic sesquiterpenes can be used as jet fuel blending agents. We predict the specific energy of combustion for these three compounds using computational approaches and we engineer microbes for the scalable production of these jet fuel candidates.

**Fig. 1:**
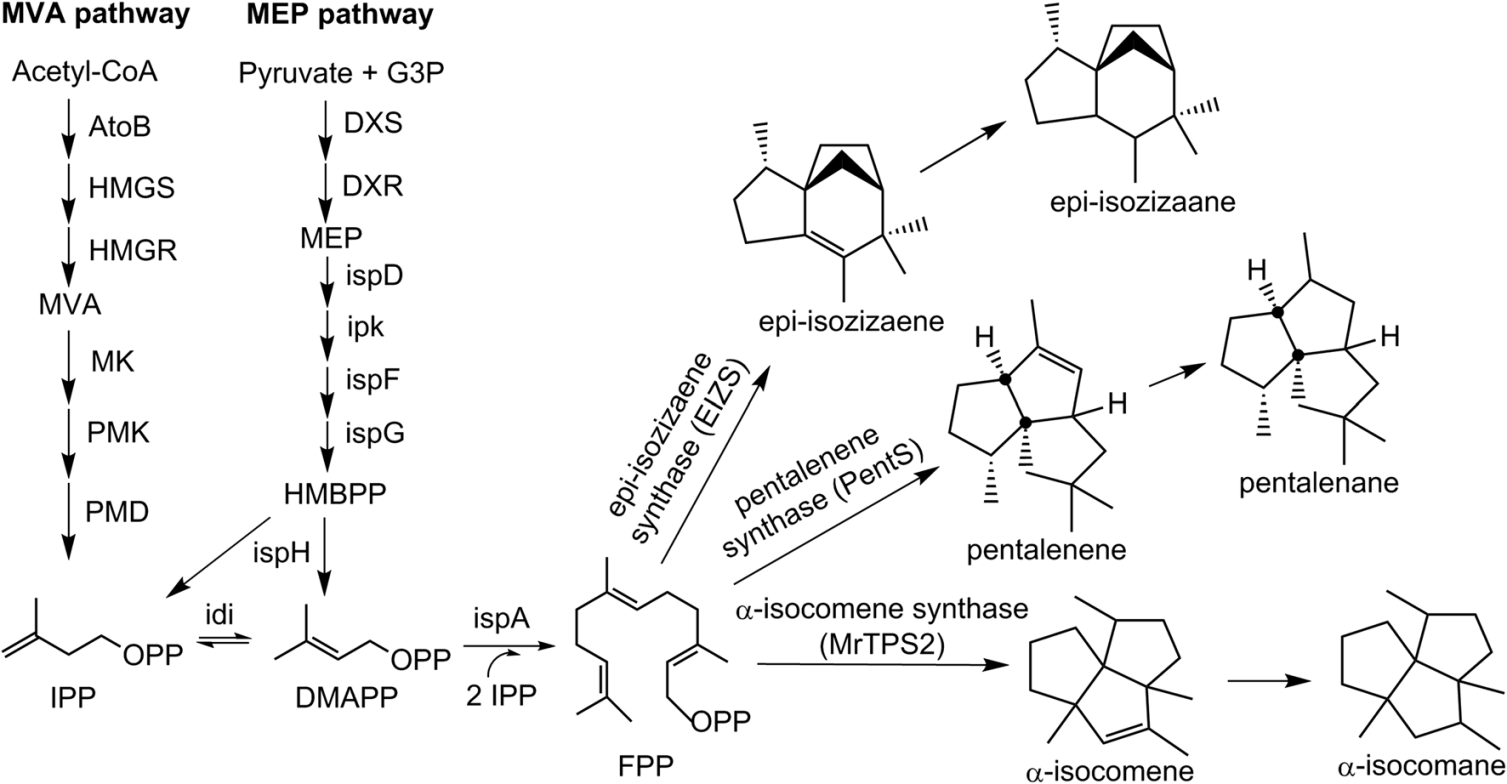
The heterologous mevalonate (MVA) pathway and the native 2-C-methyl-d-erythritol 4-phosphate (MEP) pathway for tricyclic sesquiterpene production. The heterologous MVA pathway from *S. cerevisiae* was overexpressed in *E. coli* to convert acetyl-CoA into farnesyl diphosphate (FPP). The endogenous MEP pathway consisting of 9 genes condenses pyruvate and G3P and contributes to FPP supplementation. FPP is converted into three sesquiterpenes by epi-isozizaene synthase (EIZS), pentalenene synthase (PentS) and α-isocomene synthase (MrTPS2). Pathway enzymes are *AtoB* acetoacetyl-CoA thiolase, *HMGS* HMG-CoA synthase, *HMGR* HMG-CoA reductase, *MK* mevalonate kinase, *PMK* phosphomevalonate kinase, *PMD* mevalonate diphosphate decarboxylase, *DXS* 1-deoxy-d-xylulose 5-phosphate synthase, *DXR* 1-deoxy-d-xylulose 5-phosphate reductoisomerase, *ispD* 4-diphosphocytidyl-2C-methyl-d-erythritol synthase, *ipk* 4-diphosphocytidyl-2-C-methyl-d-erythritol kinase, *ispF* 2-C-methyl-d-erythritol 2,4-cyclodiphosphate synthase, *ispG* 1-hydroxy-2-methyl-2-(E)-butenyl 4-diphosphate synthase, *ispH* 1-hydroxy-2-methyl-butenyl 4-diphosphate reductase, *idi* IPP isomerase, and *ispA* FPP synthase. Pathway intermediates are *MVA* mevalonate, *G3P* glyceraldehyde 3-phosphate, *MEP* 2-C-methyl-d-erythritol 4-phosphate, *HMBPP* 1-hydroxy-2-methyl-2-(E)-butenyl 4-pyrophosphate, *IPP* isopentenyl diphosphate, *DMAPP* dimethylallyl diphosphate, and *FPP* farnesyl diphosphate

In previous research, the epi-isozizaene (EIZS), pentalenene (PentS) and α-isocomene (MrTPS2) synthases were identified [22–24], and their in vitro activities on farnesyl diphosphate (FPP) were verified. Despite these findings, little has been reported on the synthesis of these sesquiterpenes using conventional industrial hosts, such as *S. cerevisiae* and *E. coli*, to achieve reasonable product titers. Here, we expressed the three sesquiterpene synthases in *E. coli* separately to produce epi-isozizaene, pentalenene and α-isocomene. We utilized both the endogenous methylerythritol phosphate (MEP) pathway and the heterologous mevalonate (MVA) pathway for efficient FPP supply in *E. coli* (Fig. 1). We codon-optimized each of the sesquiterpene synthases and engineered their promoters to boost sesquiterpene production. To reduce production costs associated with chemical inducers and to minimize human supervision during fermentation process, we developed an inducer-free system for production of these sesquiterpenes by introducing previously identified FPP-responsive dynamic promoters [25]. Finally, we introduced the pathway for pentalenene production into *S. cerevisiae*, an important industrial host, and demonstrated production levels comparable to those achieved using *E. coli*.

## Results

### Energy calculation of hydrogenated multicyclic sesquiterpenes

Several tricyclic sesquiterpenes (C_15_) have recently been reported with cetane numbers and cold properties comparable to conventional jet fuels [13]. In addition, the rings and the strained structure of these compounds are generally considered to contribute to increased volumetric energy density.

To further verify their suitability as jet fuel precursors, we calculated the energy of combustion for the hydrogenated products of three sesquiterpenes epi-isozizaene, pentalenene and α-isocomene (epi-isozizaane, pentalenane, and α-isocomane, respectively) using Gaussian 09 software by B3LYP method [26]. First, the isodesmic reactions were designed to calculate the theoretical enthalpy of these hydrogenated compounds. Sequentially, the combustion reaction equation was set to obtain the standard enthalpy of formation and specific energy of compounds. The detailed calculation methods and steps are described in the Additional file 1. The specific energies of epi-isozizaane, pentalenane and α-isocomane were calculated as 42.584 MJ/kg, 42.609 MJ/kg and 42.783 MJ/kg, respectively, which is similar to that of the commercial jet fuel Jet A-1 (42.80 MJ/kg) [27].

In this calculation, we did not include the changing of vaporization enthalpy (*Δ*_vap_*H*_m_) of water with temperature. When the heat release of water from gas into liquid is considered, the specific energy of these compounds will increase 2.559 MJ/kg (as *Δ*_vap_*H*_m_ (100 °C) = 40.63 kJ/mol). In summary, the calculated values of combustion energy of these hydrogenated sesquiterpenes are high enough for these compounds to be promising jet fuel candidates.

### Verification of tricyclic sesquiterpene production in *E. coli*

For microbial production of the three jet fuel precursor candidates, the sesquiterpene synthases responsible for production of these sesquiterpenes were identified in the literature. Epi-isozizaene synthase (EIZS) from *Streptomyces coelicolor* [17, 28], pentalenene synthase (PentS) from *Streptomyces* UC5319 [22, 23], and α-isocomene synthase (MrTPS2) from *Matricaria recutita* (chamomile) [20] were selected among those reported terpene synthases, and expressed in *E. coli* after the gene sequences were codon-optimized to generate *coEIZS*, *coPentS* and *coMrTPS2*. We compared solubility of these codon-optimized enzymes with the original EIZS without codon-optimization as a control. All the terpene synthases tested showed clear bands on SDS-PAGE gel in the whole cell lysate and supernatant, suggesting that they are soluble and well expressed in *E. coli* (Additional file 1: Figure S1).

**Fig. 2:**
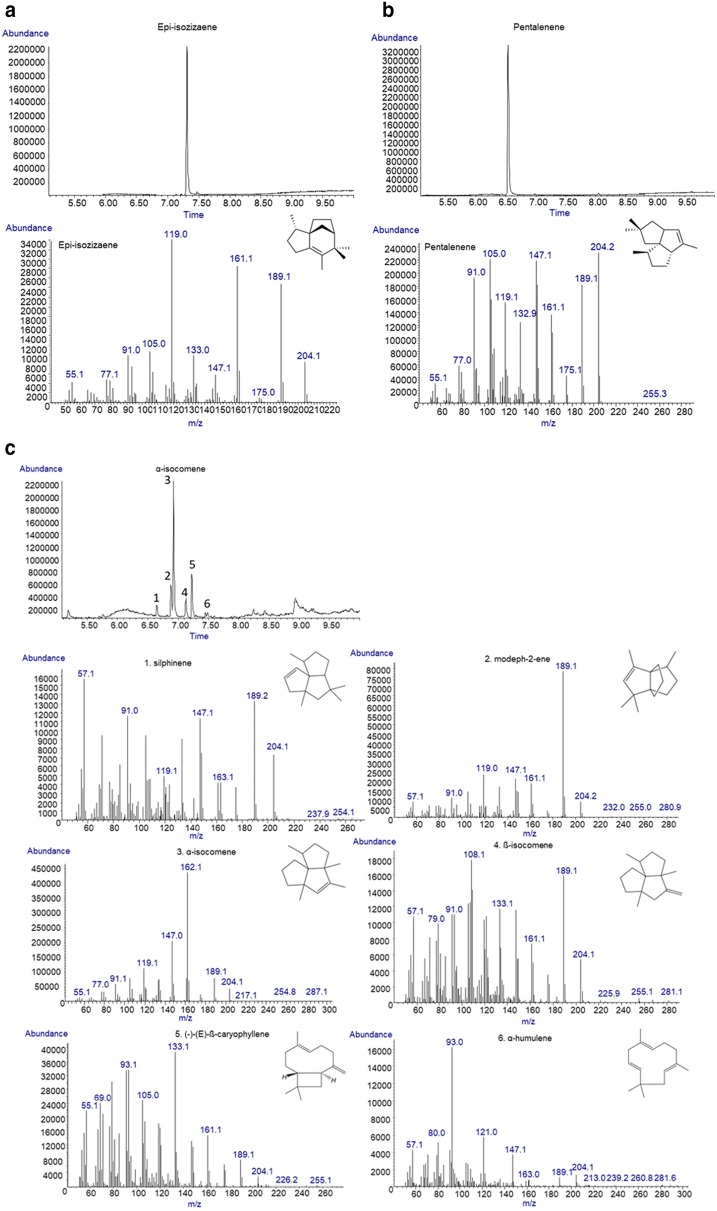
Gas chromatography–mass spectrometry (GC–MS) profile of biosynthetic sesquiterpenes. **a** Retention time and fragmentation pattern of biosynthetic epi-isozizaene (RT: 7.32 min) [17]. **b** Retention time and fragmentation pattern of produced pantalenene (RT: 6.56 min) [45]. **c** Retention time and fragmentation pattern of a broad spectrum of products from MrTPS2. The mixed products include silphinene (RT: 6.67 min), modeph-2-ene (RT: 6.90 min), α-isocomene (RT: 6.95 min), β-isocomene (RT: 7.15 min), caryophyllene (RT: 7.25 min), α-humulene (RT: 7.47 min) [20, 46]

*Escherichia coli* has an endogenous isoprenoid pathway (MEP pathway) that produces FPP, a substrate for sesquiterpene synthases, even though the natural level of FPP is quite low [29]. To confirm the in vivo activity of these three sesquiterpene synthases, we cultured strains harboring each plasmid expressing these enzymes under control of P_trc_. A decane overlay was used for in situ extraction of the product, and the collected decane layer was analyzed using GC–MS. We found that cultures expressing coEIZS and coPentS produced single products which showed fragmentation pattern of epi-isozizaene and pentalenene, respectively (Fig. 2a, b). On the other hand, the culture expressing coMrTPS2 produced a broad spectrum of compounds, including α-isocomene, silphinene, modeph-2-ene, β-isocomene, (-)-(E)-β-caryophyllene and α-humulene, with α-isocomene being the major product (Fig. 2c).

**Fig. 3:**
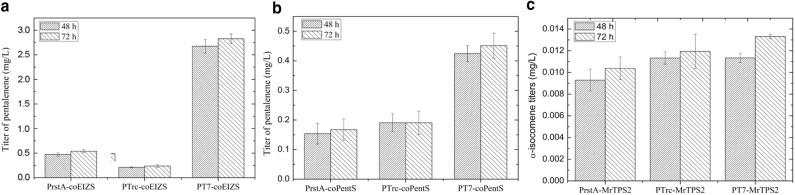
Sesquiterpene production with MEP pathway. **a** Epi-isozizaene production with strains, each harboring plasmid JBEI-15849, JBEI-15862 and JBEI-15866. **b** Pentalenene production with strains, each harboring plasmid JBEI-15863, JBEI-15867 and JBEI-15858, respectively. **c** α-Isocomene production with strains, each harboring plasmid JBEI-15864, JBEI-15865 and JBEI-15859. Titers of compounds were measured by GC–MS at 48 and 72 h, and data represent averages of three biological replicates

To enhance sesquiterpene production, we increased the expression levels of the genes encoding coEIZS, coPentS and coMrTPS2 using a stronger inducible promoter (Fig. 3). The use of the T7 promoter (P_T7_) significantly improved biosynthesis of epi-isozizaene and pentalenene compared to strains harboring a plasmid with P_trc_-driven terpene synthases, probably due to the increased terpene synthase levels. The titers of epi-isozizaene and pentalenene improved 11.8- and 2.4-fold to 2.82 mg/L and 0.45 mg/L, respectively, after 48 h. Interestingly, no significant titer improvement was observed for cells harboring the P_T7_-driven α-isocomene synthase; the titer was still more than an order of magnitude lower than those of pentalenene and epi-isozizaene, which was probably due to low enzyme activity of coMrTPS2.

### High titer sesquiterpene production using the heterologous mevalonate pathway

**Fig. 4:**
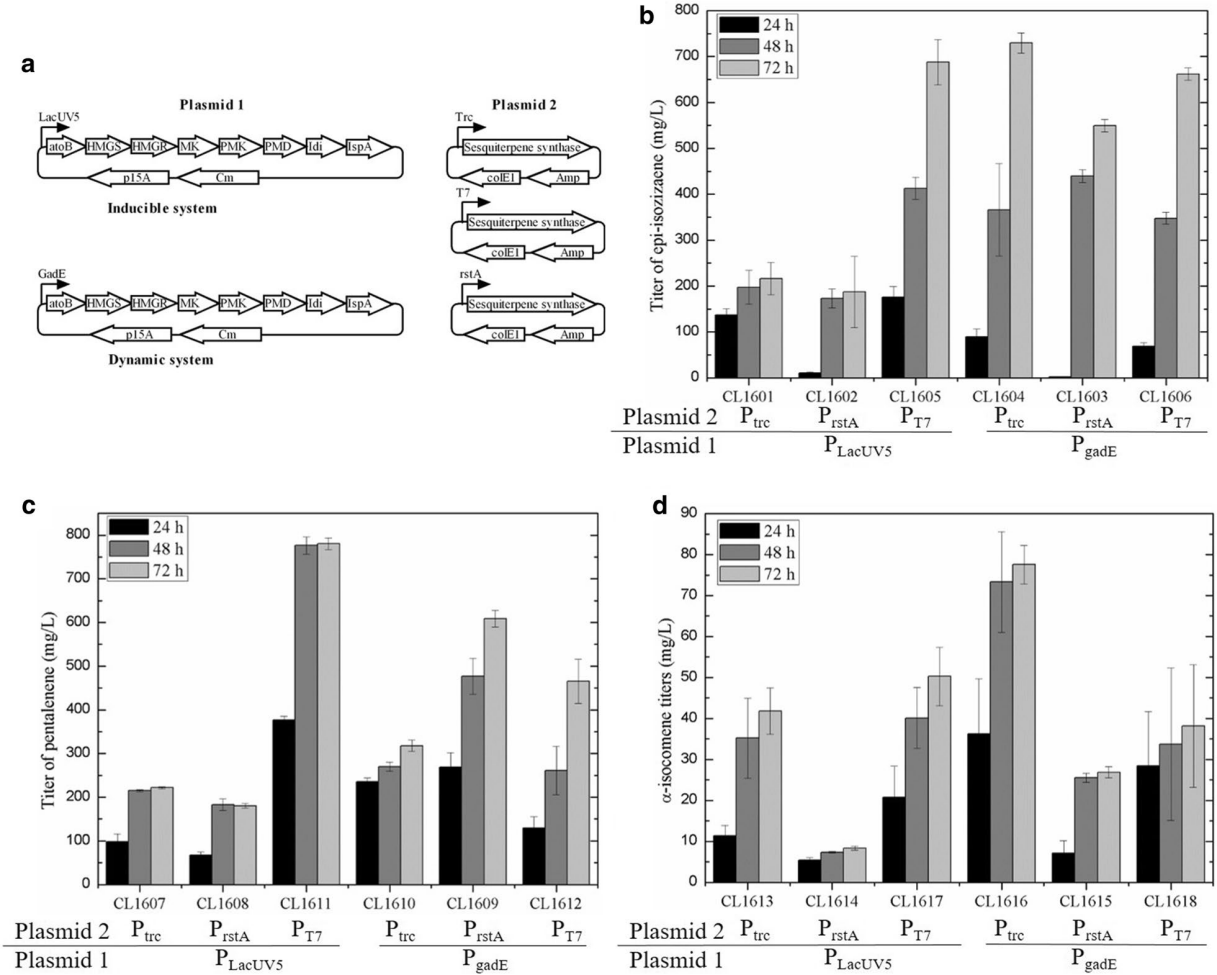
Sesquiterpene production with the MVA pathway and promoter engineering. **a** Plasmid architecture. A two-plasmid system for sesquiterpene production. Plasmid 1 contains MVA pathway genes producing FPP from acetyl-CoA either under a P_lacUV5_ (inducible) or an FPP-responsive promoter P_gadE_ (dynamic) with a medium-copy origin (p15A) and chloramphenicol resistance gene. Plasmid 2 contains a sesquiterpene synthase gene under either P_trc_, P_T7_ or an FPP-responsive promoter P_rstA_ with a high-copy origin (colE1) and ampicillin resistance gene. **b** Epi-isozizaene production. **c** Pentalenene production. **d** α-isocomene production. Titers of compounds were measured at 24, 48 and 72 h. Promoters for both plasmid 1 and plasmid 2 are listed to describe strains as well as strain names in *x*-axis. Data represent averages of three biological replicates with standard deviation as error bars

The native MEP pathway is tightly controlled by endogenous regulation and may not supply sufficient FPP for high titer sesquiterpene biosynthesis [8]. We have observed a generally low production level of sesquiterpenes with the native MEP pathway in *E. coli* when terpene synthases are overexpressed. Previous studies showed that terpene production can be dramatically improved by introducing a heterologous mevalonate (MVA) pathway as the flux to FPP is significantly increased with the heterologous pathway [7, 30]. The eight-enzyme MVA pathway converts acetyl-CoA into FPP [30], and a system in which the genes encoding those enzymes are harbored on a single medium-copy plasmid (“plasmid 1”) and the terpene synthase is harbored on a high-copy plasmid (“plasmid 2”) is well established (Fig. 4a) [31].

We first used an inducible promoter system to express MVA pathway enzymes and the terpene synthase. We co-transformed the “plasmid 1” containing MVA pathway genes under IPTG-inducible promoter (P_lacUV5_) and “plasmid 2” containing the terpene synthase under control of either P_trc_ or P_T7_ into *E. coli* DH1. This resulted in the “inducible” sesquiterpene production systems: strain CL1601 and CL1605 for epi-isozizaene production, CL1607 and CL1611 for pentalenene production, CL1613 and CL1617 for α-isocomene production (Table 1).

**Table 1:** Description of *E. coli* base strains, plasmids and gene clusters used in this study

Plasmid reference	Plasmid name	Relevant genotype	References	
JBEI-2704	placUV5-MevT-MBIS	p15A, CmR	[44]	
JBEI-2872	pgadE-MevT-MBIS	p15A, CmR	[25]	
JBEI-15857	pTrc99a-EIZS	ColE1(pBR322) ori, AmpR	This study	
JBEI-15862	pTrc99a-coEIZS	ColE1(pBR322) ori, AmpR	This study	
JBEI-15867	pTrc99a-coPentS	ColE1(pBR322) ori, AmpR	This study	
JBEI-15865	pTrc99a-coMrTPS2	ColE1(pBR322) ori, AmpR	This study	
JBEI-15856	pTrc99a-his-EIZS	ColE1(pBR322) ori, AmpR, his tag	This study	
JBEI-15855	pTrc99a-his-coEIZS	ColE1(pBR322) ori, AmpR, his tag	This study	
JBEI-15854	pTrc99a-his-coPentS	ColE1(pBR322) ori, AmpR, his tag	This study	
JBEI-15853	pTrc99a-his-coMrTPS2	ColE1(pBR322) ori, AmpR, his tag	This study	
JBEI-15849	prstA-coEIZS	ColE1(pBR322) ori, AmpR	This study	
JBEI-15863	prstA-coPentS	ColE1(pBR322) ori, AmpR	This study	
JBEI-15864	prstA-coMrTPS2	ColE1(pBR322) ori, AmpR	This study	
JBEI-15866	pBbE7a-coEIZS	ColE1(pBR322) ori, AmpR	This study	
JBEI-15858	pBbE7a-coPentS	ColE1(pBR322) ori, AmpR	This study	
JBEI-15859	pBbE7a-coMrTPS2	ColE1(pBR322) ori, AmpR	This study	
JBEI-15861	pRSLeu2d-coPentS	2μ ori, pBR322 ori, Leu2d	This study	

Strain name	Description	References	
CL1601	JBEI-2704 + JBEI-15862	This study	
CL1602	JBEI-2704 + JBEI-15849	This study	
CL1603	JBEI-2872 + JBEI-15849	This study	
CL1604	JBEI-2872 + JBEI-15862	This study	
CL1605	JBEI-2704 + JBEI-15866	This study	
CL1606	JBEI-2872 + JBEI-15866	This Study	
CL1607	JBEI-2704 + JBEI-15867	This study	
CL1608	JBEI-2704 + JBEI-15863	This Study	
CL1609	JBEI-2872 + JBEI-15863	This study	
CL1610	JBEI-2872 + JBEI-15867	This study	
CL1611	JBEI-2704 + JBEI-15858	This study	
CL1612	JBEI-2872 + JBEI-15858	This study	
CL1613	JBEI-2704 + JBEI-15865	This study	
CL1614	JBEI-2704 + JBEI-15864	This study	
CL1615	JBEI-2872 + JBEI-15864	This study	
CL1616	JBEI-2872 + JBEI-15865	This study	
CL1617	JBEI-2704 + JBEI-15859	This study	
CL1618	JBEI-2872 + JBEI-15859	This study	
CL1619	*S. cerevisiae* EPY300 harboring JBEI-15861	This study	

In general, expression of the MVA pathway resulted in considerable production of target sesquiterpenes. As shown in Fig. 4, the titers of epi-isozizaene and pentalenene were significantly increased to 215.8 mg/L and 221.7 mg/L after 72 h in P_trc_ strains CL1601 and CL1607 (i.e., P_trc_ is driving sesquiterpene synthases), respectively, compared to those without the MVA pathway. When P_T7_ was driving the sesquiterpene synthases in strains CL1605 and CL1612, the titers increased by 3.2- and 3.5-fold over those strains under P_trc_, respectively. For α-isocomene biosynthesis, the titer increase achieved by swapping terpene synthase promoters was much smaller than values observed for epi-isozizaene. The α-isocomene titer was 41.8 mg/L in strain CL1613 when P_trc_ was used to drive expression of MrTPS2; the titer increased to 50.2 mg/L in strain CL1617 in which P_T7_ drives MrTPS2. In general, these results confirm that a heterologously expressed MVA pathway is much more efficient in sesquiterpene production than the endogenous MEP pathway. Regarding significant titer improvements observed when terpene synthases were driven by P_T7_, we reasoned that the strong pulling effect by high terpene synthase levels driven by P_T7_ may provide a driving force for the entire pathway by depleting the intermediate metabolite as substrates for the corresponding enzymes as well as by relieving the inhibition of mevalonate kinase by FPP. From the targeted proteomics data (Additional file 1: Figure S10) and the metabolomics data (Additional file 1: Figure S9), we could see that the level of coEIZS in strain CL1605 was much higher than that in strain CL1601, and the FPP level in strain CL1605 was lower than that in CL1601. This negative correlation indicates that the high terpene synthase levels achieved by stronger, P_T7_-driven *coEIZS* gene expression may significantly rewire pathway flux towards the conversion of FPP into epi-isozizaene.

### Introduction of the FPP-responsive dynamic system for sesquiterpene production

The overproduction of FPP using an inducible heterologous MVA pathway substantially improved sesquiterpene titers. Inducible promoters, however, cannot regulate the intracellular FPP level in response to changes of cell growth or environmental conditions, and they may lead to suboptimal intracellular FPP concentrations, which can be toxic to cells [25]. In addition to the lack of dynamic control, the conventional inducible promoters require both the use of expensive chemicals such as IPTG as inducer and human supervision for induction timing. These make the inducible systems less economically feasible in a working industrial setting. To address these challenges, two FPP-responsive promoters P_*gadE*_ and P_*rstA*_ were previously found to be useful in creating an auto-inducible isoprenoid production system [25]. The *gadE* promoter (P_*gadE*_) down-regulates expression of genes under its control when the FPP level is high, while the *rstA* promoter (P_*rstA*_) up-regulates gene expression at high FPP levels. Therefore, using P_*gadE*_ for expression of FPP-producing enzymes and P_*rstA*_ for expression of FPP-utilizing enzymes results in a dynamic system to control FPP levels and produce sesquiterpenes at high titers [25]. Guided by these findings, we systematically studied the effects of P_*gadE*_ and P_*rstA*_ on tricyclic sesquiterpene production.

We first constructed plasmids expressing each terpene synthase under control of P_*rstA*_ and confirmed their expression by testing terpene production using the endogenous MEP pathway. We observed the production of 0.54 mg/L epi-isozizaene, 0.19 mg/L pentalenene and 0.01 mg/L α-isocomene without adding any inducer; these levels are comparable to the production via the strain with terpene synthases under IPTG-inducible P_trc_ (Fig. 3). Then, the IPTG-inducible MVA pathway was co-expressed with each of three terpene synthases under control of P_*rstA*_ (strains CL1602, CL1608, and CL1614 for epi-isozizaene, pentalenene, and α-isocomene production, respectively). We compared the sesquiterpene production of these strains with that from strains with terpene synthases under IPTG-inducible promoters (P_trc_ and P_T7_) (Fig. 4). The specific titers of epi-isozizaene, pentalenene, and α-isocomene in strain CL1602, CL1608, and CL1614 were 125.9 mg/L, 180.4 mg/L, and 8.4 mg/L, respectively. The titers of epi-isozizaene and pentalenene were slightly lower than those of strains with terpene synthases under control of the IPTG-inducible P_trc_. They were significantly lower than those from strains that used P_T7_ for terpene synthase expression. It is probably due to much lower terpene synthase level in the strains with P_*rstA*_ than those in the strain with P_T7_. The titers of α-isocomene, however, were not straightforward to explain in the same way as α-isocomene producing strains show more complex products profile as shown in Fig. 2c probably due to the promiscuity of α-isocomene synthase. But the general trend could still be explained by much lower terpene synthase level in the strains with P_rstA_ than those in the strain with P_T7_.

An FPP-responsive promoter P_*gadE*_ was introduced for the MVA pathway gene expression to build a complete dynamic system for sesquiterpene production as previously reported [25]. We prepared sets of six strains for each sesquiterpene synthase by combining two MVA pathway plasmids (either IPTG-inducible or FPP-responsive) and three terpene synthase plasmids (under either IPTG-inducible (P_trc_ and P_T7_) or FPP-responsive (P_*rstA*_) promoters) as shown in Table 1; CL1601–CL1606 for epi-isozizaene production, CL1607–CL1612 for pentalenene production, and CL1613–CL1618 for α-isocomene production.

In contrast to the IPTG-inducible MVA pathway for epi-isozizaene (CL1601, CL1602, CL1605) and pentalenene (CL1607, CL1608, CL1611) production, the use of dynamic promoter for the expression of the MVA pathway resulted in product titers that were less dependent on the type of promoter driving the terpene synthase (Fig. 4b, c). When the IPTG-inducible MVA pathway was used, there was a significant improvement in terpene production with strains containing the P_T7_-driven terpene synthases over strains with P_trc_ or P_*rstA*_ driving epi-isozizaene and pentalenene synthase expression (Fig. 4). This suggests that the cyclization of FPP to sesquiterpene products by the terpene synthase is the primary bottleneck in the pathway, and the activity or expression level of terpene synthase is a primary determinant of the titer when the IPTG-inducible MVA pathway was used. In contrast, when the dynamic promoter was used for FPP production via the MVA pathway for epi-isozizaene (CL1604, CL1603, CL1606) and pentalenene (CL1610, CL1609, CL1612) production, there was smaller fluctuation in terpene production among strains with three different promoters driving terpene synthase expression (Fig. 4b, c). This result makes sense as the dynamic promoter (P_*gadE*_) can regulate the MVA pathway gene expression and, therefore, the metabolic flux to maintain the FPP level balanced. The combination of P_*gadE*_-MVA with P_*rstA*_- or P_trc_-driven sesquiterpene synthase enabled the cell to balance FPP production and consumption and, therefore, to auto-regulate the levels of this toxic intermediate as proven by the measured intracellular FPP levels (Additional file 1: Figure S9). The combination of P_T7_-driven sesquiterpene synthases with P_*gadE*_-MVA also achieved a balanced flux and considerable epi-isozizaene titer, but it is considered mainly due to the high expression level of coEIZS (Additional file 1: Figure S10). Because the FPP-responsive dynamic system auto-regulates the production pathway in case of FPP depletion or FPP over-accumulation, the dynamically controlled system can improve sesquiterpene production through balanced FPP levels.

For epi-isozizaene production, three strains with dynamically regulated MVA pathway showed comparable titers to the highest producing inducible system (CL1605) after 48 h, but their production level at 24 h was about half of those with the inducible MVA pathway (Fig. 4b). This trend, however, does not apply to pentalenene-producing system. For pentalenene production, the highest titer was achieved using strain CL1611 which has the MVA pathway under control of P_lacUV5_ and coPentS under control of P_T7_. The inducible system with P_T7_ driving the terpene synthase (CL1611) showed a significantly higher titer than the other pentalenene strains. With the exception of CL1611, the titers at 24 h were more than twofold higher in strains with a dynamically regulated MVA pathway than in those with the inducible MVA pathway. It is an interesting result and probably caused by the difference in the activity and the availability of two terpene synthases. Since FPP is a toxic intermediate, the FPP consumption efficiency by terpene synthase affects the balance of the entire pathway as well as the titer. The kinetics of EIZS and PentS are not available (and it would be out of scope of this work), but the differences in the activity and the relative amount of soluble enzyme shown in the SDS-PAGE results of these two terpene synthases (Additional file 1: Figure S1) would be responsible for the different trends in FPP accumulation and production formation in these strains.

Strain CL1609 had the full dynamic system and produced the second highest pentalenene titer and the highest OD_600_ (Additional file 1: Figure S8). This indicates more sensitive stress-responsive dynamic regulation in CL1609. The cell growth of CL1610 and CL1612 (Additional file 1: Figure S7) restricted compound biosynthesis. For α-isocomene production, the worst performer was strain CL1614 containing P_*lacUV5*_-MVA and P_*rstA*_-coMrTPS2, probably due to the low level of terpene synthase expression under weak promoter (P_*rstA*_) leading to intracellular FPP accumulation and the MVA pathway expression unbalanced. This unbalance was avoided in strain CL1615 by the dynamic regulation of the MVA pathway under P_*gadE*_, and this strain showed a decent level of product. Interestingly, the best producer was strain CL1616, which contains the dynamically controlled MVA pathway and the IPTG-inducible terpene synthase (P_trc_-coMrTPS2). A similar system with a stronger IPTG-inducible promoter driving the terpene synthase (CL1618) showed a comparable titer at 24 h, but the production did not improve much after 24 h and showed a large standard deviation. These observations suggest FPP production in CL1618 was not well balanced for terpene production. It is possible that the initial consumption of FPP by highly abundant terpene synthase in the early production stage triggered rapid FPP biosynthesis under dynamic promoter (P_*gadE*_). This, however, may have resulted in a rapid accumulation of FPP and have disrupted the MVA pathway through un-identified process to halt FPP production. In summary, when an inducible promoter (P_lacUV5_) drives expression of the genes encoding the MVA pathway, we observed that the combination with P_T7_-driven terpene synthase, which is expected to have the highest terpene synthase level, achieved the highest sesquiterpene production.

Combinations of the dynamic promoter (P_*gadE*_)-driven MVA pathway and gene *coEIZS* or *coPentS* driven by either inducible or dynamic promoters resulted in reasonably high epi-isozizaene and pentalenene titers regardless of terpene synthase promoter strength, showcasing the advantages of a dynamic system over an inducible system. Although the fully dynamic system containing both FPP-responsive promoters (P_*gadE*_ and P_*rstA*_) did not lead to the highest production, large amounts of sesquiterpenes were still obtained for epi-isozizaene (549.7 mg/L) and pentalenene (608.5 mg/L).

### Omics-aided evaluation of IPTG-inducible and dynamic promoter systems

We investigated changes in MVA pathway enzyme expression and metabolite production in response to the dynamic promoters compared to the IPTG-inducible system using various omics technologies. We chose epi-isozizaene production strains with 4 different combinations of promoters (strains CL1601 and CL1604 with P_trc_-coEIZS, and CL1605 and CL1606 with P_T7_-coEIZS; Table 1) and collected their metabolomics (Additional file 1: Figure S9) and proteomics (Additional file 1: Figure S10) data at six time points over 48 h. The concentrations of acetyl-CoA decreased after 4 h in all four strains, and MVA concentrations, which are the highest among the pathway metabolites monitored, increased at early production phases. MVA levels then slowly decreased in all four strains, but MVA concentration reached much higher level in strains with P_T7_ (CL1605 and CL1606) than the other strains before it started to decrease. Interestingly, while the MVA levels in strain CL1605 were almost 20 times higher than that in strain CL1601 at the late fermentation phase, other metabolites such as Mev-P, IPP/DMAPP, and FPP showed similar levels in CL1601 and CL1605. From the proteomics analysis shown in Additional file 1: Figure S10, we found that CL1601 expressed the lowest levels of MVA pathway proteins overall among these four strains, which may explain the poor production titer observed from this strain. Compared to CL1601, the enzyme levels were significantly higher in CL1605. Especially for proteins AtoB, HMGS and HMGR, strain CL1605 expressed about 5–10 times more enzymes than CL1601. This may explain the markedly higher levels of MVA in CL1605. It is interesting to observe that the use of strong promoter (P_T7_) for terpene synthase gene expression improved both the level of coEIZS enzyme as intended and the expression of upstream pathway enzymes. In our speculation, this is related to the deficiency of FPP in early stage (Additional file 1: Figure S9) due to high level of terpene synthase. FPP is an essential metabolite for *E. coli* growth and when the strain experiences a deficit of FPP, it pushes the pathway to produce more FPP for survival. As a result, the first three enzymes of the MVA pathway were expressed more. However, the next two enzymes (MK and PMK) levels remained quite low as previously observed [44] and this may have limited the conversion of MVA to the next intermediates.

We observed dynamic changes in intracellular FPP concentrations from strain CL1604 harboring P_*gadE*_-MevT-MBIS (JBEI-2872) and P_trc_-CoEIZS plasmids (JBEI-15862) (Additional file 1: Figure S9F). In this case, the FPP level decreased in the first 2 h, increased between 2 and 6 h, decreased again between 6 and 8 h, then eventually kept slowly accumulating in the cell. This observation is consistent with previous findings [25]. However, we did not observe a similar trend in strain CL1606, which harbors JBEI-2872 and JBEI-15866 expressing epi-isozizaene synthase under a strong promoter (P_T7_), where FPP concentration was constantly maintained at very low level. This behavior might have resulted from the strong promoter (P_T7_) expressing excessive epi-isozizaene synthase, which depleted intracellular FPP in CL1606. The low FPP level de-represses P_*gadE*_ and makes it behave constitutively in CL1606. This is consistent with generally higher levels of the first three enzymes, which are directly influenced by the promoter strength, in strain CL1606 over strain CL1604 (Additional file 1: Figure S10).

### Large-scale production, purification and NMR characterization of the biosynthetic sesquiterpenes

Epi-isozizaene and pentalenene are not commercially available in large quantities. Experimental testing of these compounds for jet fuel specification, however, requires a large quantity of these compounds. Therefore, it is required to demonstrate a larger-scale fermentation of these sesquiterpenes producers to achieve this goal. To demonstrate scalability and the downstream purification process, we performed a large-scale production of these jet fuel precursors in 4-L flasks with batch culture (TB medium with 0.4% glycerol) using the inducer-free dynamic *E. coli* strains CL1603 and CL1609 for epi-isozizaene and pentalenene, respectively. During large-scale production, the cultures were overlayed with 20% nonane instead of decane to facilitate sesquiterpene extraction, as the boiling temperature of nonane (151 °C) is lower than that of decane or dodecane (174.1 °C or 216.2 °C, respectively), which makes it easier to recover the product from the overlay by evaporation of the solvent.

About 0.8 L and 0.5 L of nonane were collected from the epi-isozizaene biosynthesis cultures and the pentalenene biosynthesis cultures, respectively, using a separatory funnel. The products were recovered from the nonane overlay by a series of purification steps as described in “Methods” section. We used NMR to identify the product and to check its purity (i.e., the specificity of terpene synthase toward the expected product) and confirmed these two compounds with the purities over 90% and assigned their NMR spectra (Additional file 1: Figures S2–S5).

Finally, we prepared about 1 mL epi-isozizaene and 0.66 mL pentalenene with over 90% purity from large-scale batch cultures (4 L for epi-isozizaene and 2.5 L for pentalenene), which contains 1.08 g epi-isozizaene and 0.66 g pentalenene. The recovery efficiencies were about 49% and 43%, respectively, when considering the production titer from GC analysis.

### Sesquiterpene production in yeast

**Fig. 5:**
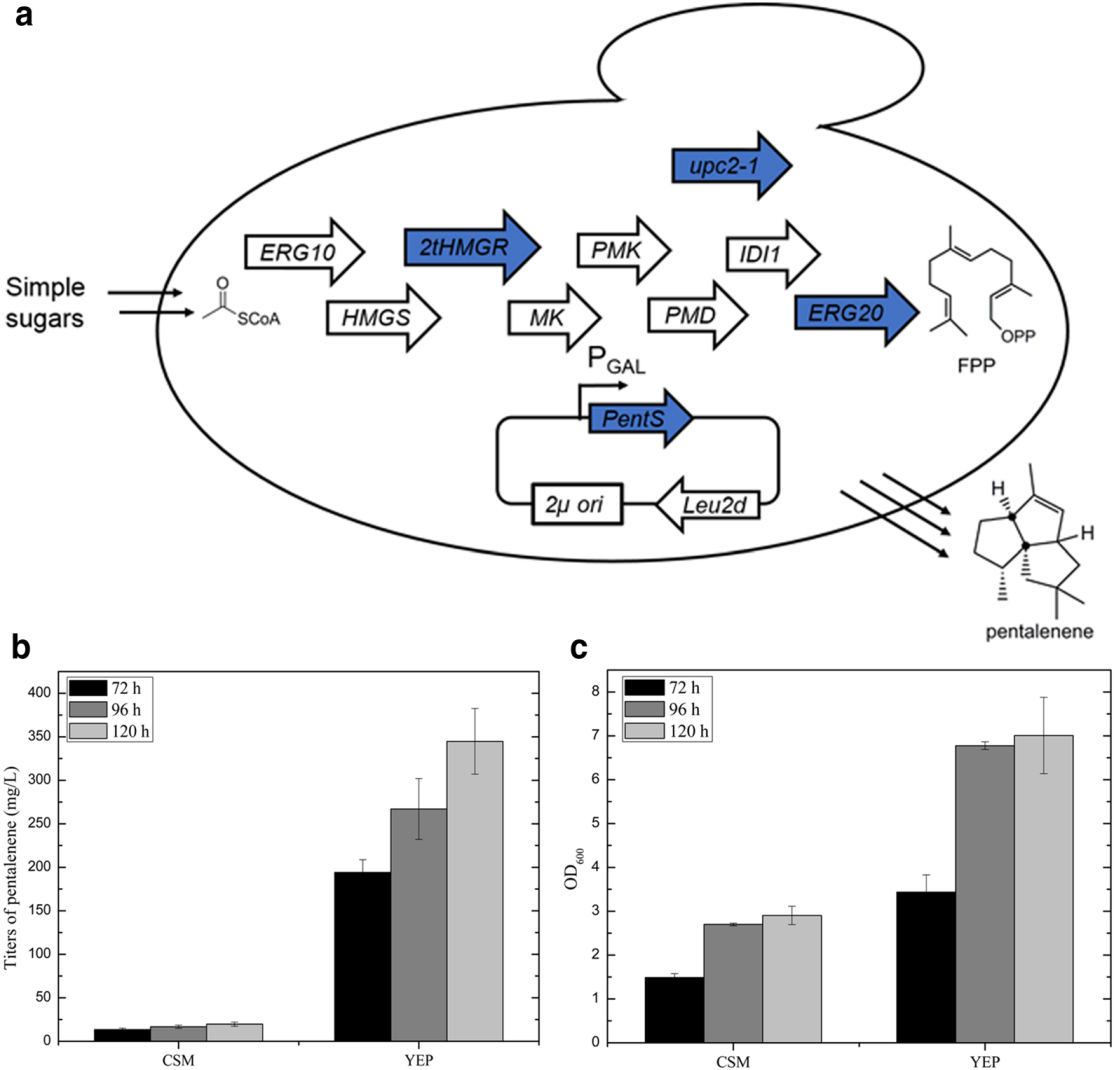
Pentalenene production in *S. cerevisiae*. **a** The *S. cerevisiae* host engineered for the production of antimalarial precursor amorphadiene was re-engineered to produce pentalenene by overexpressing pentalenene synthase (PentS) in a plasmid. *ERG10* acetyl-CoA acetyltransferase, *IDI1* isoprenyl diphosphate isomerase, *ERG20* farnesyl disphosphate synthase. Genes in blue arrows (*upc2*-*1*, *tHMGR*, *ERG20* and *PentS*) are overexpressed. **b** Production titer and **c** cell growth (OD_600_) with CSM medium and YEP medium supplemented with 1.8% galactose/0.2% glucose

The Baker’s yeast, *Saccharomyces cerevisiae*, has been the host of choice for industrial production of various bio-products and widely used for terpene biosynthesis. Therefore, in addition to production in *E. coli* we sought to demonstrate the production of one of these sesquiterpenes, pentalenene in *S. cerevisiae* (Fig. 5).

We employed a previously reported host strain engineered for high FPP production (EPY300) [32] (Fig. 5a). To efficiently convert FPP to pentalenene, we introduced the codon-optimized gene *coPentS* under the control of a galactose promoter on a high-copy plasmid (2 μ) containing the auxotrophic Leu2d marker, as previously used to achieve high levels of amorphadiene [33]. We used both defined CSM medium and non-selective, rich, mixed carbon YEP medium supplemented with 1.8% galactose/0.2% glucose for culturing the yeast cells. Consequently, higher cell density and production were achieved using the rich medium. After 120 h, 344.7 mg/L pentalenene was obtained in mixed carbon YEP medium, which was 17.5-fold higher than that in CSM medium (Fig. 5b).

## Discussion

In this work, we proposed three tricyclic sesquiterpenes as promising jet fuel precursors. The calculated combustion energies of the target sesquiterpenes show positive results for their use as jet fuels. Additionally, we engineered microbes to produce these jet fuel precursors at high titers to demonstrate the potential of microbial jet fuel manufacturing. In particular, we developed an inducer-free system using previously identified FPP-responsive promoters and obtained a respectable amount of sesquiterpenes, allowing future application to the industrial fermentation process.

The engineered MVA pathway is a very efficient FPP supply platform for sesquiterpene biosynthesis and the increased FPP flux drives the implementation of FPP-responsive promoters (P_*gadE*_ and P_*rstA*_) to render higher production titers by relieving the toxicity from FPP accumulation, as previously reported [25]. This dynamic setting, however, did not always result in higher titer compared to the inducible system (with P_lacUV5_ and P_T7_ promoters) used in this study, and this implies that there are more factors that affect efficiency of the dynamically regulated system and it may require additional adjustment to achieve balanced enzyme expression and optimized pathway performance. Finally, we demonstrated the production of one of these sesquiterpenes in *S. cerevisiae*, a microbial host that is widely used for biofuel production. Much work including terpene synthase enzyme engineering (for a better activity and specificity) and culture medium and process optimization (for scale-up) would be needed to improve the titer, yield, and productivity of these sesquiterpenes in *S. cerevisiae*.

Tricyclic sesquiterpenes are expected to have several advantages over the already commercialized acyclic bio-jet fuel precursor, farnesene (or Biofene^®^ from Amyris) as versatile feedstocks to jet fuels. First, they have higher densities than farnesene (around 1.0 g/mL vs 0.81 g/mL of farnesene), which deliver higher volumetric net heats of combustion [13, 34]. Considering small increases in volumetric energy density can have major impacts on profitability of airlines, especially for long haul flights, higher density fuel would be preferred over lower density fuels. Also, while farnesene has four double bonds and requires four molar equivalents of dihydrogen to generate the actual fuel molecule (saturated hydrocarbons, farnesanes), tricyclic sesquiterpenes have only one double bond and require just one dihydrogen molecule per fuel molecule, making the refining process less expensive.

In the past decade, the farnesene biosynthetic pathway has been extensively studied and optimized [35–37]. Currently, a production of $2.2/kg has been achieved and the estimated price will become even lower in the near future [38]. Since tricyclic sesquiterpenes and farnesene share a large portion of their biosynthetic pathways, we expect that an engineering strategy similar to that used to produce farnesene could be applied to achieve high productivity and yield of tricyclic terpenes. In this study, we have achieved only about a gram scale production to demonstrate the production, isolation, and characterization. This amount, however, might be not quite enough for any jet fuel specification testing as it requires much larger amounts of fuel candidates to test standard jet fuel specification such as ASTM D1655. The successful demonstration of the downstream purification processes we presented in this report would be a fundamental work for producing large quantities of sesquiterpene compounds in future. More studies including production scale-up will be also needed for further validation of these new jet fuel compounds.

## Conclusions

Sesquiterpenes and sesquiterpene-derived molecules are promising alternatives to aviation fuels. In the present study, we proposed tricyclic sesquiterpene compounds, epi-isozizaene, pentalenene and α-isocomene as jet fuel precursors to expand the scope of bio-jet fuels. The sesquiterpene synthases were introduced into *E. coli* with either endogenously or heterologously expressed isoprenoid pathways, and the production pathway was engineered to increase the product titer. IPTG-inducible and dynamically controlled systems were compared for sesquiterpene production; proteomics and metabolomics analysis of these systems explain the different behaviors of various strains. In addition, we generated a yeast strain for one of the jet fuel targets, pentalenene, to explore an alternate host for large scale fermentation. In summary, the engineered strains produced tricyclic sesquiterpenes at high titers with 727.9 mg/L epi-isozizaene, 780.3 mg/L pentalenene and 77.5 mg/L α-isocomene in *E. coli* in a batch culture with 10 g/L glucose, and 344 mg/L pentalenene in *S. cerevisiae* with YPG medium. The current study will provide a solid ground toward sustainable alternatives to high energy density fuels for the long-term global energy demand.

## Methods

### Plasmids and strains construction

All plasmids and strains used in this study are available on the JBEI public registry (https://public-registry.jbei.org) and listed in Table 1 along with a brief description of production strains. *E. coli* DH10B was used for plasmid construction, and *E. coli* DH1 and *E. coli* DH1 (DE3) were used as hosts for terpene production and protein expression. Plasmids containing eight genes for the mevalonate pathway (*atoB*, *hmgs*, *hmgr*, *mk*, *pmk*, *pmd*, *idi*, *ispA*) were introduced into *E. coli* host, enabling production of FPP from acetyl-CoA. Five genes (*hmgs*, *hmgr*, *mk*, *pmk*, *pmd*) originated from *S. cerevisiae* and three genes (*atoB*, *idi*, *ispA*) are native *E. coli* genes. For sesquiterpene production in *E. coli*, four terpene synthase genes [the original *EIZS* from *Streptomyces* and three *E. coli* codon-optimized genes, *coEIZS*, *coPentS* and *coMrTPS2*, which were amplified from gBlock templates synthesized by IDT (Iowa, USA)] were cloned into pTrc99 at *Nco*I and *Xba*I sites, to yield the plasmids JBEI-15857, JBEI-15862, JBEI-15867 and JBEI-15865, respectively. To insert an N-terminal His-tag for protein purification, PCR was performed using the primers that included a His-tag sequence and the PCR product was cloned into pTrc99 vector yielding plasmids JBEI-15856, JBEI-15855, JBEI-15854 and JBEI-15853, respectively. Genes *coEIZS*, *coPentS* and *coMrTPS2* were cloned into vector pPrstA-RFP [25] via the isothermal assembly method [39] using a commercial Gibson Assembly^®^ Master Mix kit (New England Biolabs) to yield plasmids prstA-coEIZS, prstA-coPentS and prstA-coMrTPS2, respectively. Plasmid pBbE7a-coEIZS, pBbE7a-coPentS and pBbE7a-coMrTPS2 were also constructed using vector pBbA7a-RFP between *Nde*I and *Xho*I sites. For pentalenene production in *S. cerevisiae*, *coPentS* was cloned into vector pRSLeu2d [31] between the *Nhe*I and *Xho*I sites. All the promoter strength was indicated by RFP florescence as shown in Additional file 1: Figure S7.

### Sesquiterpene production in *E. coli*

For MVA pathway expression, *E. coli* DH1 was co-transformed with an MVA pathway plasmid JBEI-2704 (for the IPTG-inducible system) or JBEI-2872 (for the dynamic system) and a plasmid harboring one of each terpene synthase gene. Pre-cultures of *E. coli* strains were diluted 1:100 into EZ rich defined medium (Teknova, Hollister, CA) containing 10 g/L glucose as carbon source and ampicillin (100 µg/mL) and chloramphenicol (30 µg/mL) for plasmid maintenance. The cell cultures were grown at 37 °C until OD_600_ reached ~ 0.6, and induced with 0.5 mM IPTG while shaking at 30 °C; 20% decane was added for in situ product extraction. At 24 h, 48 h and 72 h time points, 10 μL of the decane layer was taken and diluted into 990 μL of ethyl acetate for GC–MS analysis. Caryophyllene was used as an internal standard for the production of epi-isozizaene and pentalenene, while pentalenene was used as internal standard for the production of α-isocomene since the strain with MrTPS2 also produces detectable amount of caryophyllene.

### Detection of sesquiterpenes with GC–MS

The decane fraction from the culture medium was analyzed by GC–MS (Thermo Trace Ultra with PolarisQ MS) with a TR-5MS column (30 m × 0.25 mm ID × 0.25 µm film) using the following conditions: inlet at 250 °C, 1.1 mL min^−1^ constant flow, transfer line at 300 °C, ion source at 200 °C, scan *m/z* 50–300. Oven: 100 °C for 4 min, ramp at 30 °C min^−1^ to 250 °C, hold for 1 min. Samples of 1 μL were injected into the GC. The products were analyzed in total ion monitoring mode and selective ion monitoring mode (*m/z* 204).

### Large-scale production, purification, and characterization of the biosynthetic sesquiterpenes

For the preparation of sesquiterpene compounds epi-isozizaene and pentalenene, the *E. coli* strains for sesquiterpene production were cultured at 30 °C for 72 h after induction. One liter of Terrific Broth (Difco) with 4% glycerol was used for batch production in a 4-L flask overlayed with 200 mL of nonane to facilitate sesquiterpene extraction. The nonane overlay was collected from the culture medium using a separatory funnel and dried over sodium sulfate. Nonane was evaporated under reduced pressure, and the crude sesquiterpenes were loaded onto a silica gel column for column chromatography using hexanes as the mobile phase. The product containing fractions were pooled and the solvent was evaporated under reduced pressure. The product was obtained as colorless oil and further purified by preparative silver ion thin-layer chromatography (Ag-TLC) for nuclear magnetic resonance (NMR) analysis. Silver nitrate was adsorbed onto thin-layer silica by methods described previously [40]. TLC plates (500 µm layer thickness) were sprayed with an AgNO_3_ solution (5% w/v in methanol), dried at 110 °C for 10 min and allowed to cool at room temperature directly before use. Plates were developed in hexane and the pure product was dissolved in n-pentane and concentrated under reduced pressure. The structure of the isolated epi-isozizaene and pentalenene dissolved in CDCl_3_ was analyzed by 1H and 13C NMR using a 500 MHz NMR spectrometer (Bruker DRX-500). NMR spectrum of biosynthetic epi-isozizaene: ^1^H NMR (600 MHz, CDCl_3_): δ 2.22 (ddp, *J* = 17.0, 9.1, 1.2 Hz, 1H), 2.13–2.03 (m, 1H), 1.83 (dd, *J* = 7.4, 5.3 Hz, 1H), 1.80–1.76 (m, 2H), 1.76–1.71 (m, 1H), 1.62–1.54 (m, 1H), 1.48 (dd, *J* = 10.5, 5.3 Hz, 1H), 1.43 (t, *J* = 1.5 Hz, 3H), 1.40 (d, *J* = 10.0, 1.9 Hz, 1H), 1.38–1.34 (m, 1H), 1.26–1.21 (m, 1H), 1.18 (tdd, *J* = 11.5, 3.4, 2.1 Hz, 1H), 1.00 (s, 3H), 0.98 (s, 3H), 0.92 (d, *J* = 6.4 Hz, 3H) (Additional file 1: Figure S2). ^13^C NMR (151 MHz, CDCl_3_): δ 143.0, 127.5, 52.7, 47.2, 40.5, 39.7, 37.0, 32.5, 28.7, 28.4, 27.3, 25.1, 24.4, 14.1, 12.9 (Additional file 1: Figure S3). NMR spectrum of biosynthetic pentalenene: ^1^H NMR (600 MHz, CDCl_3_): δ 5.15 (h, *J* = 1.6 Hz, 1H), 2.66 (ddh, *J* = 9.3, 4.5, 2.1 Hz, 1H), 2.54 (d, *J* = 9.4 Hz, 1H), 1.86–1.80 (m, 1H), 1.78 (ddd, *J* = 12.5, 6.1, 3.0 Hz, 1H), 1.73 (dd, *J* = 13.1, 1.0 Hz, 1H), 1.62 (h, *J* = 1.4 Hz, 4H), 1.37–1.23 (m, 4H), 1.18 (ddd, *J* = 12.5, 5.1, 0.9 Hz, 1H), 0.98 (s, 3H), 0.98 (s, 3H), 0.90 (d, *J* = 7.1 Hz, 3H) (Additional file 1: Figure S4). ^13^C NMR (151 MHz, CDCl_3_): δ 140.5, 129.5, 64.7, 62.0, 59.3, 48.9, 46.8, 44.5, 40.5, 33.5, 29.9, 29.1, 27.5, 17.0, 15.5 (Additional file 1: Figure S5).

### Metabolite analysis and targeted proteomics

All metabolites were analyzed by liquid chromatography–mass spectrometry (LC–MS; Agilent Technologies 1200 Series Rapid Resolution HPLC system and Agilent Technologies 6210 time-of-flight mass spectrometer (TOF–MS) (Agilent Technologies, Santa Clara, CA, USA) on a SeQuant^®^ ZIC^®^-pHILIC column (150 mm length, 2.1-mm internal diameter, and 5-µm particle size). Targeted proteomics was performed using an Agilent 1290 liquid chromatography system coupled to an Agilent 6460 QQQ mass spectrometer (Agilent Technologies, Santa Clara, CA) via selected reaction monitoring (SRM) at the same points of the samples of metabolites. The sample preparation and detailed method of metabolite analysis and targeted proteomics are presented in Additional file 1: Supplementary Methods.

### Pentalenene production in *S. cerevisiae*

Pre-cultures of *S. cerevisiae* strain harboring the plasmid pRsLeu2d-coPentS were used to inoculate at an OD_600_ of 0.05 into 5 mL of Complete Supplemental Mixture (CSM; MB Biomedicals, Solon, OH) medium or rich mixed carbon yeast extract peptone (YEP; 1% yeast extract, 2% peptone) medium supplemented with 1.8% galactose/0.2% glucose and overlayed with 10% decane. At 72 h, 96 h and 120 h, 10 μL decane layer was sampled and diluted 100 times into ethyl acetate with caryophyllene as the internal standard. For pentalenene quantification, the samples were analyzed by GC–MS as described above.

### Energy calculation

The thermodynamic parameters of the two compounds were calculated by means of density functional theory (DFT) method using Gaussian 09 software. All the DFT calculations were performed using the hybrid B3LYP method with the 6-311+G(2d,p) basis set level [26, 41], which can provide a nice balance between cost and accuracy, and is known to perform very well for the prediction of geometries of compounds. The specific energy was calculated following the steps of the isodesmic reaction to get the standard enthalpy of formation, as well as considering the combustion reaction equation to get the enthalpy values of combustion. Isodesmic reactions have been found to be a more accurate computational approach than atomization enthalpy calculations or heat of atomization calculations as demonstrated previously in calculating enthalpy of formation of formaldehyde [42]. It has also been used in other bond energy calculation [43]. The detailed calculation methods and steps are shown in the Additional file 1: Supplementary Methods.

## Additional file

**Additional file 1.** Supplementary figures and supplementary methods. **Figure S1:** Purification and characterization of sesquiterpene synthases. **Figures S2 and S3:** ^1^H and ^13^C NMR spectrum of biosynthetic epi-isozizaene. **Figures S4 and S5:** ^1^H and ^13^C NMR spectrum of biosynthetic pentalenene. **Figure S6:** Epi-isozizaene production in *E. coli* DH1 via the native MEP pathway catalyzed by the epi-isozizaene synthase. **Figure S7:** The RFP florescence indicating promoter strength. **Figure S8:** OD_600_ of *E. coli* strains for sesquiterpenes production. **Figure S9:** Metabolite analysis. **Figure S10:** Targeted proteomic analysis of pathway enzymes.
