# Supplementary material for: Renewable production of high density jet fuel precursor sesquiterpenes from Escherichia coli
Source: Biotechnol Biofuels. 2018 Oct 20;11:285. doi: 10.1186/s13068-018-1272-z (PMC6195743; doi:10.1186/s13068-018-1272-z)
Supplement: Supplementary file 1 — Additional file 1. Supplementary figures and supplementary methods. Figure S1: Purification and characterization of sesquiterpene synthases. Figures S2 and S3: 1H and 13C NMR spectrum of biosynthetic epi-isozizaene. Figures S4 and S5: 1H and 13C NMR spectrum of biosynthetic pentalenene. Figure S6: Epi-isozizaene production in E. coli DH1 via the native MEP pathway catalyzed by the epi-isozizaene synthase. Figure S7: The RFP florescence indicating promoter strength. Figure S8: OD600 of E. coli strains for sesquiterpenes production. Figure S9: Metabolite analysis. Figure S10: Targeted proteomic analysis of pathway enzymes. [file 13068_2018_1272_MOESM1_ESM.docx]

Figure S1. Purification and characterization of sesquiterpene synthases. (A) SDS-PAGE for protein analysis. From left to right line: 1-Marker, 2-Control, 3-EIZS-W, 4-EIZS-S, 5-COEIZS-W, 6-COEIZS-S, 7-MrTPS2-W, 8-MrTPS2-S, 9-PentS-W,10-PentS-S. (B) SDS-PAGE for the purified protein MrTPS2. As the MrTPS2 protein was too dilute in crude cell lysate, we further enriched this protein. From left to right line: 1-MrTPS2-W, 2-MrTPS2-S, 3-purified elution buffer, 4 and 5-concentrated protein, 6-Marker, 7-Control-W, 8-Control-S. W stands for whole cell, and S stands for supernatant. The soluble protein EIZS and coEIZS have a size of 41.4 kDa, protein coPentS appears at 38 kDa, and coMrTPS2 appears at 63.4 kDa.


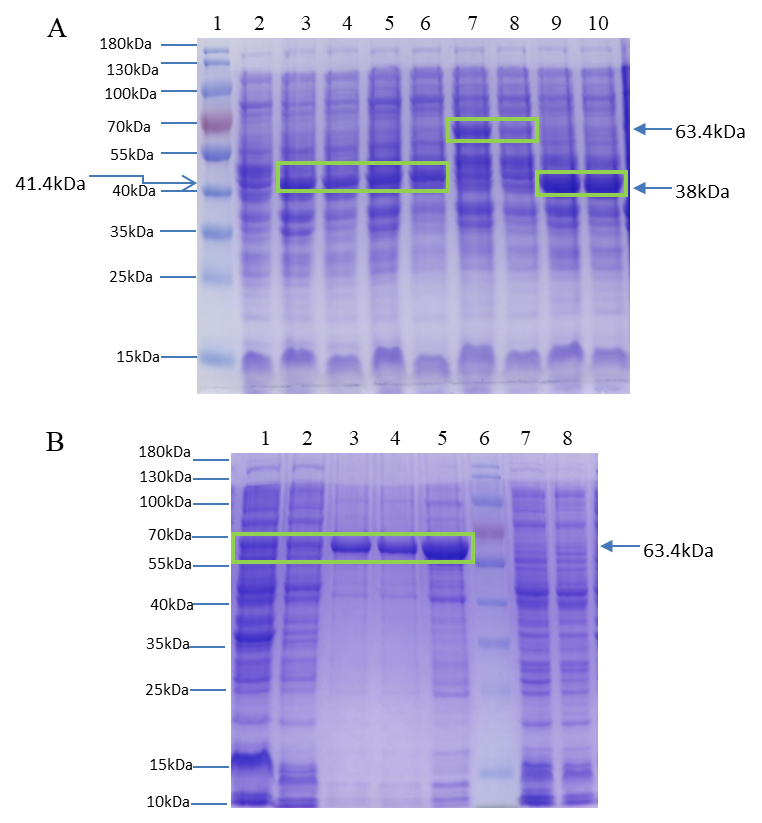


Figure S2. ^1^H NMR spectrum of biosynthetic epi-isozizaene. ^1^H NMR (600 MHz, CDCl_3_): δ 2.22 (ddp, J = 17.0, 9.1, 1.2 Hz, 1H), 2.13 – 2.03 (m, 1H), 1.83 (dd, J = 7.4, 5.3 Hz, 1H), 1.80 – 1.76 (m, 2H), 1.76 – 1.71 (m, 1H), 1.62 – 1.54 (m, 1H), 1.48 (dd, J = 10.5, 5.3 Hz, 1H), 1.43 (t, J = 1.5 Hz, 3H), 1.40 (d, J = 10.0, 1.9 Hz, 1H), 1.38 – 1.34 (m, 1H), 1.26 – 1.21 (m, 1H), 1.18 (tdd, J = 11.5, 3.4, 2.1 Hz, 1H), 1.00 (s, 3H), 0.98 (s, 3H), 0.92 (d, J = 6.4 Hz, 3H).


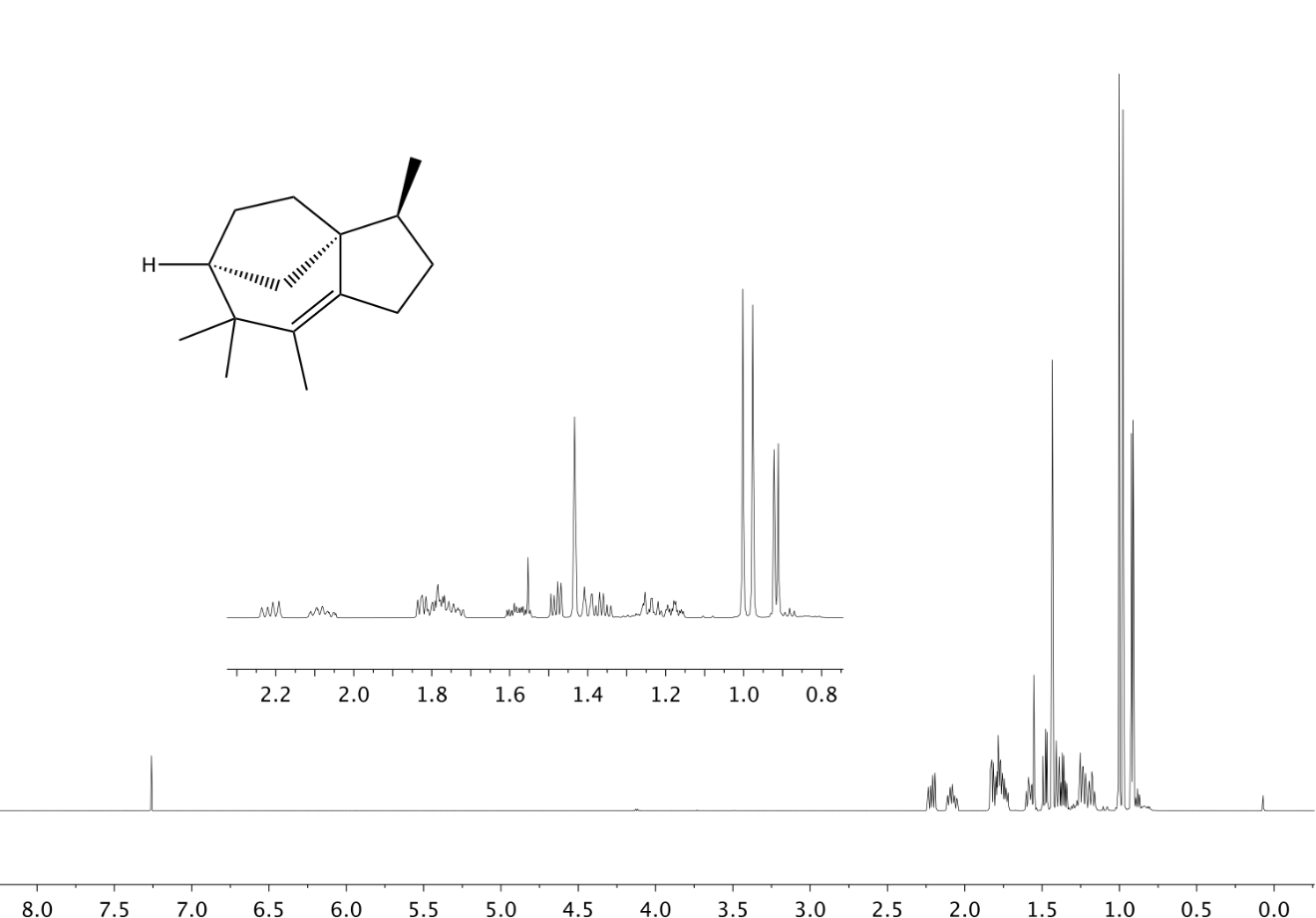


Figure S3. ^13^C NMR spectrum of biosynthetic epi-isozizaene. ^13^C NMR (151 MHz, CDCl_3_): δ 143.0, 127.5, 52.7, 47.2, 40.5, 39.7, 37.0, 32.5, 28.7, 28.4, 27.3, 25.1, 24.4, 14.1, 12.9.


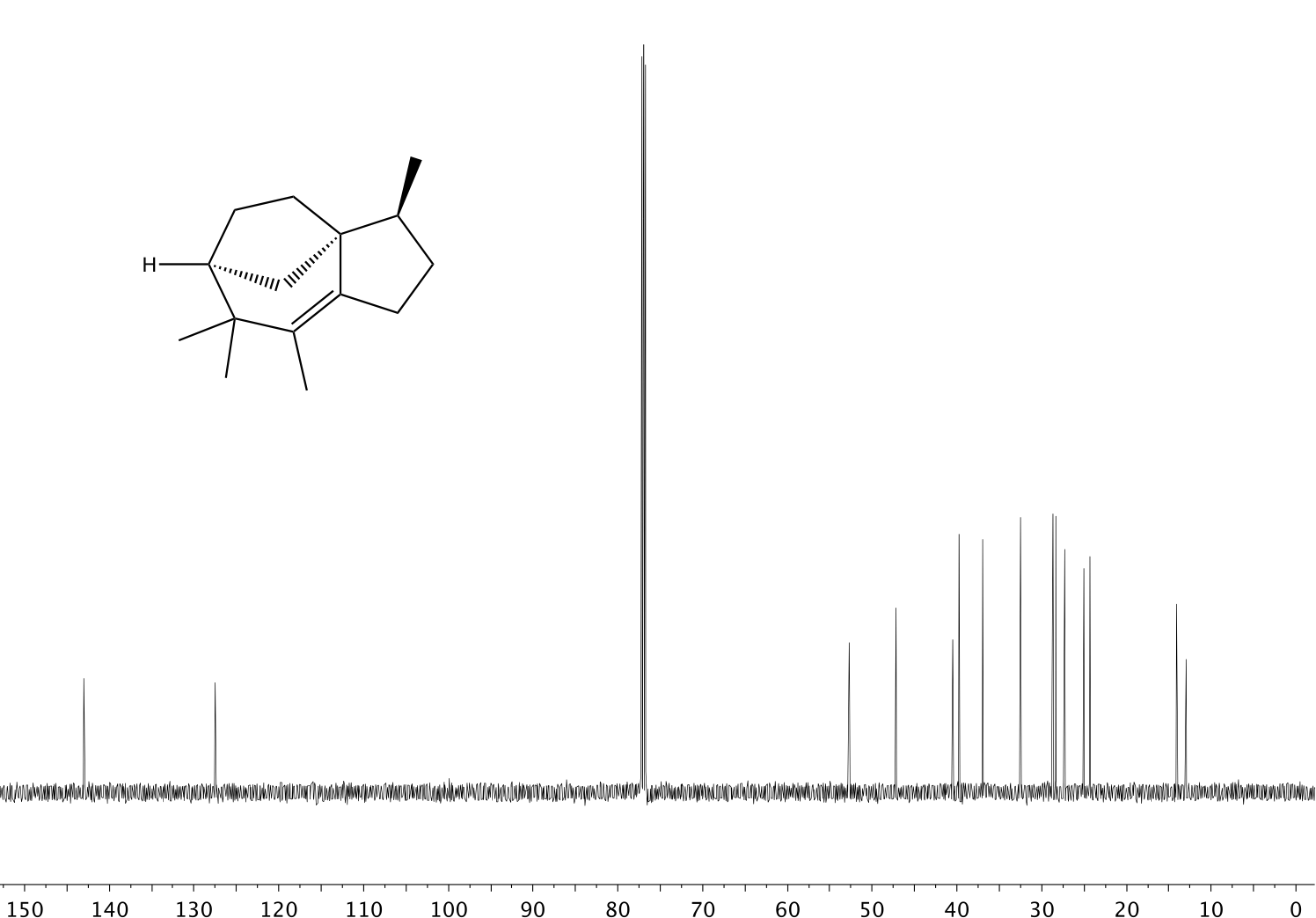


Figure S4. ^1^H NMR spectrum of biosynthetic pentalenene. ^1^H NMR (600 MHz, CDCl_3_): δ 5.15 (h, J = 1.6 Hz, 1H), 2.66 (ddh, J = 9.3, 4.5, 2.1 Hz, 1H), 2.54 (d, J = 9.4 Hz, 1H), 1.86 – 1.80 (m, 1H), 1.78 (ddd, J = 12.5, 6.1, 3.0 Hz, 1H), 1.73 (dd, J = 13.1, 1.0 Hz, 1H), 1.62 (h, J = 1.4 Hz, 4H), 1.37 – 1.23 (m, 4H), 1.18 (ddd, J = 12.5, 5.1, 0.9 Hz, 1H), 0.98 (s, 3H), 0.98 (s, 3H), 0.90 (d, J = 7.1 Hz, 3H).


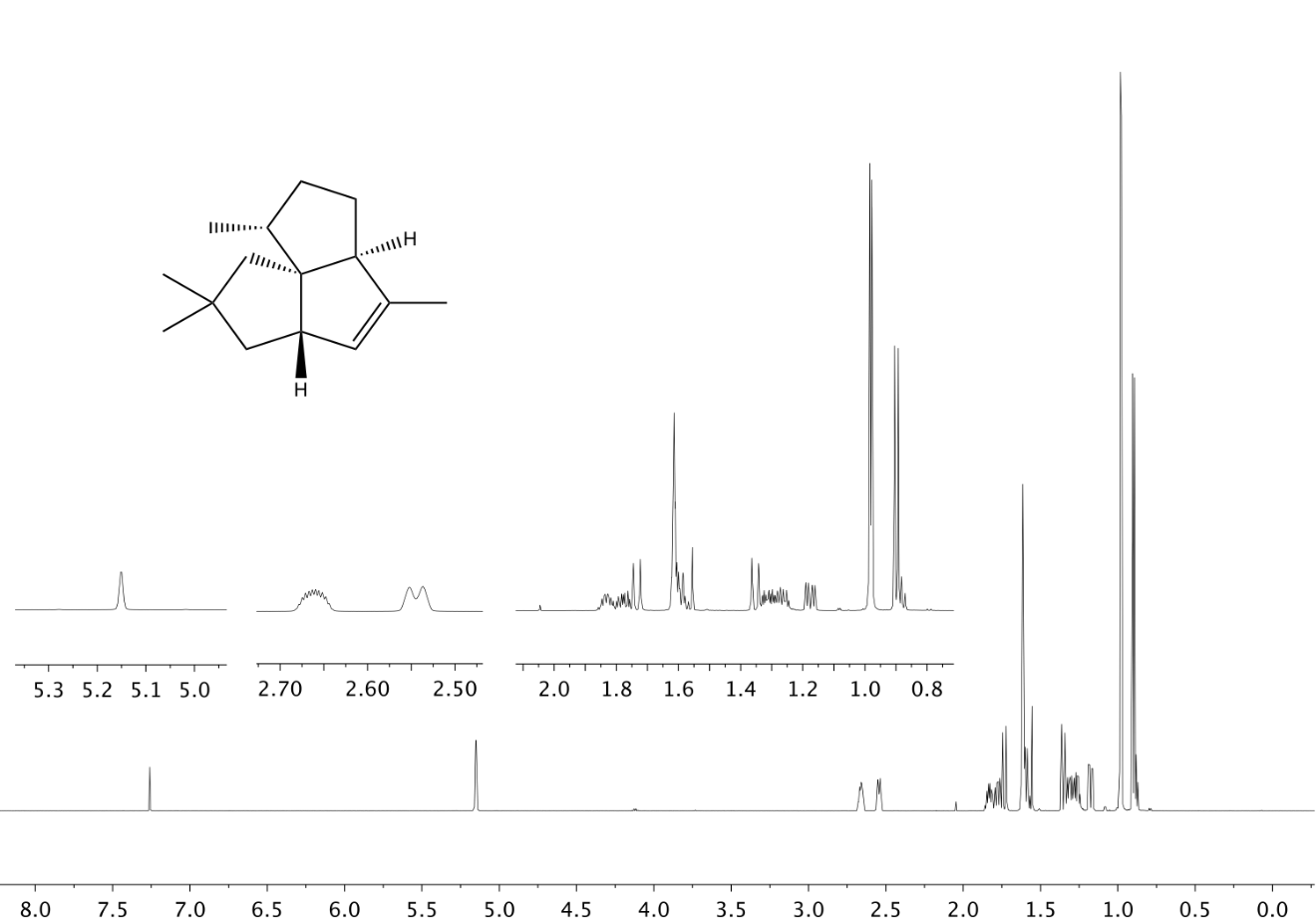


Figure S5. ^13^C NMR spectrum of biosynthetic pentalenene. ^13^C NMR (151 MHz, CDCl_3_): δ 140.5, 129.5, 64.7, 62.0, 59.3, 48.9, 46.8, 44.5, 40.5, 33.5, 29.9, 29.1, 27.5, 17.0, 15.5.


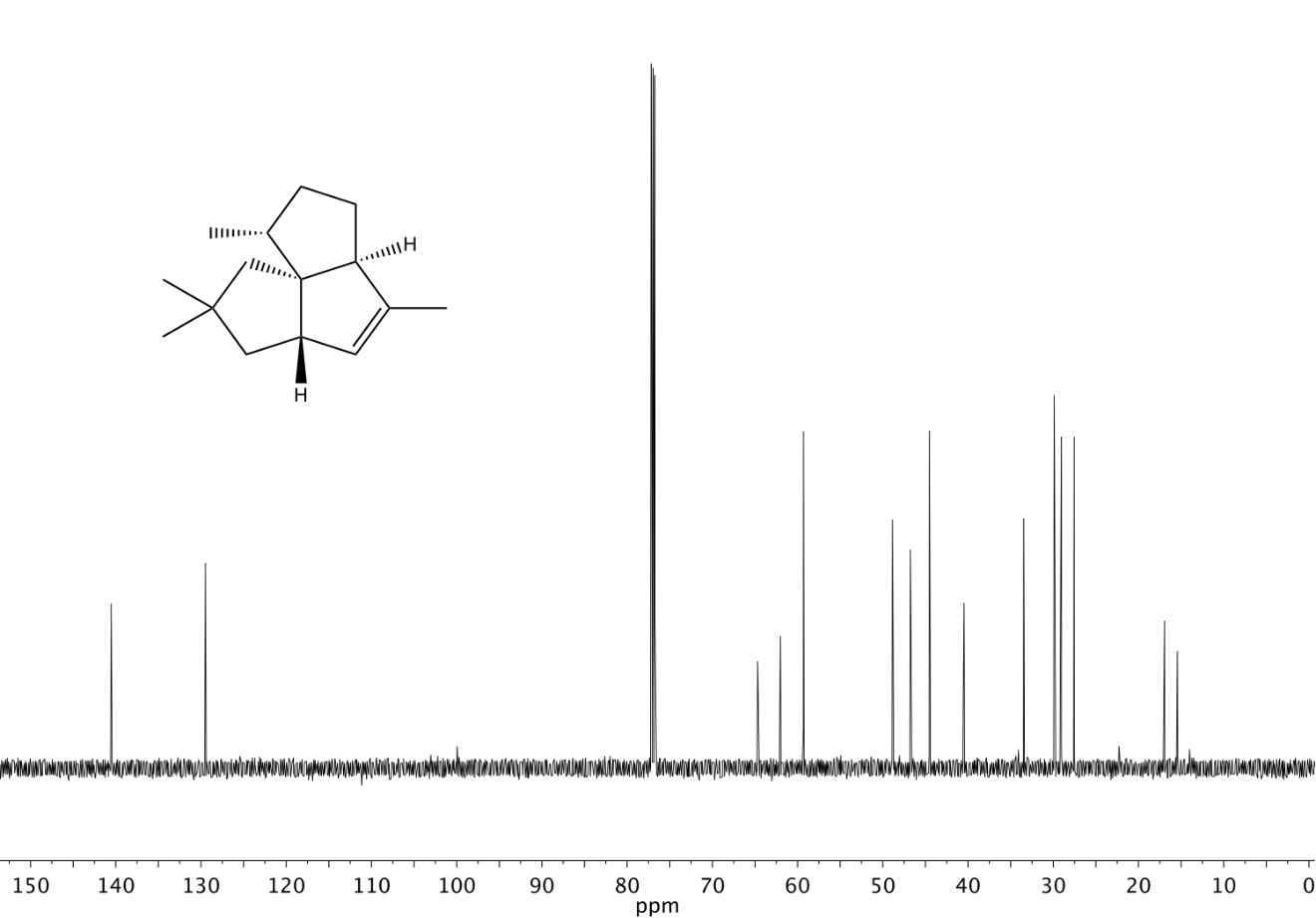


Figure S6. Epi-isozizaene production in *E. coli* DH1 via the native MEP pathway catalyzed by the epi-isozizaene synthase from original gene (*EIZS*) and codon-optimized gene (*coEIZS*).





Figure S7. The RFP florescence indicating promoter strength. The RFP gene under the control of different promoters (i.e. Trc, rstA, T7, lacUV5, and gadE) were cloned into vectors with colE1 ori and Ampicillin resistance. The IPTG-inducible promoter strains (induced by 0.5 mM IPTG) and the constitutive promoter strains were all cultured for 20 hours at 30°C in LB medium before the RFP florescence was quantified and normalized to cell growth.





Figure S8. OD_600_ of *E. coli* strains for sesquiterpenes production. (A) Strain CL1601 ~ CL1606 for epi-isozizaene production, (B) CL1607 ~ CL1612 for pentalenene production, and (C) CL1613 ~ CL1618 for α-isocomene production.


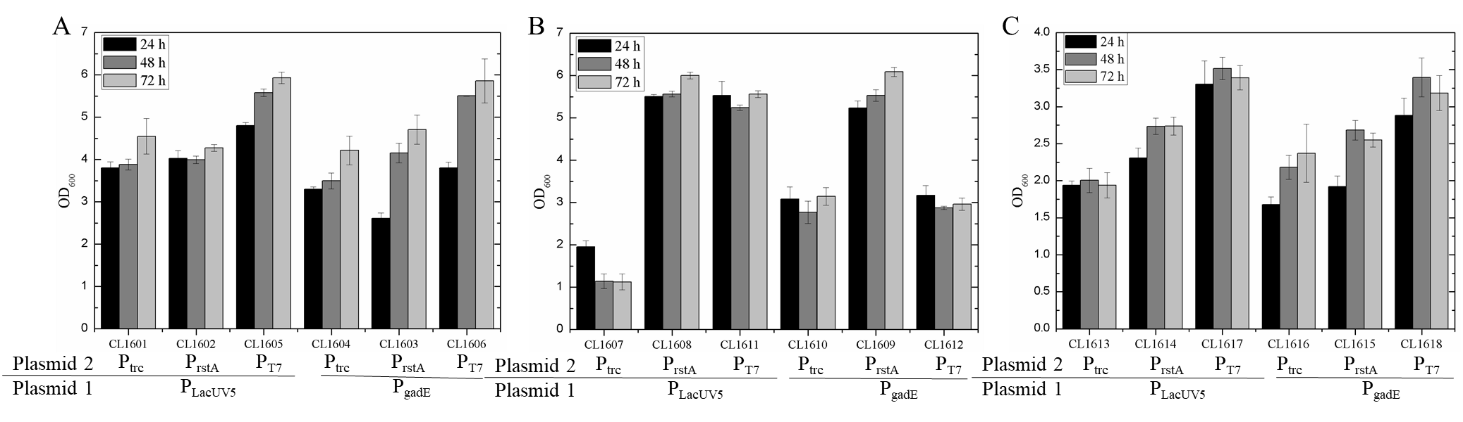


**Figure S9.** Metabolite analysis of the inducible control system strain CL1601 and CL1605, and dynamic control system strain CL1604 and CL1606 from induction time (0 h) to 48h. The concentrations of metabolite (A) Acetyl CoA, (B) HMG-CoA, (C) MVA, (D) Mev-P, (E) IPP/DMAPP and (F) FPP were analyzed by LC-MS. Data represent averages from three biological replicates.


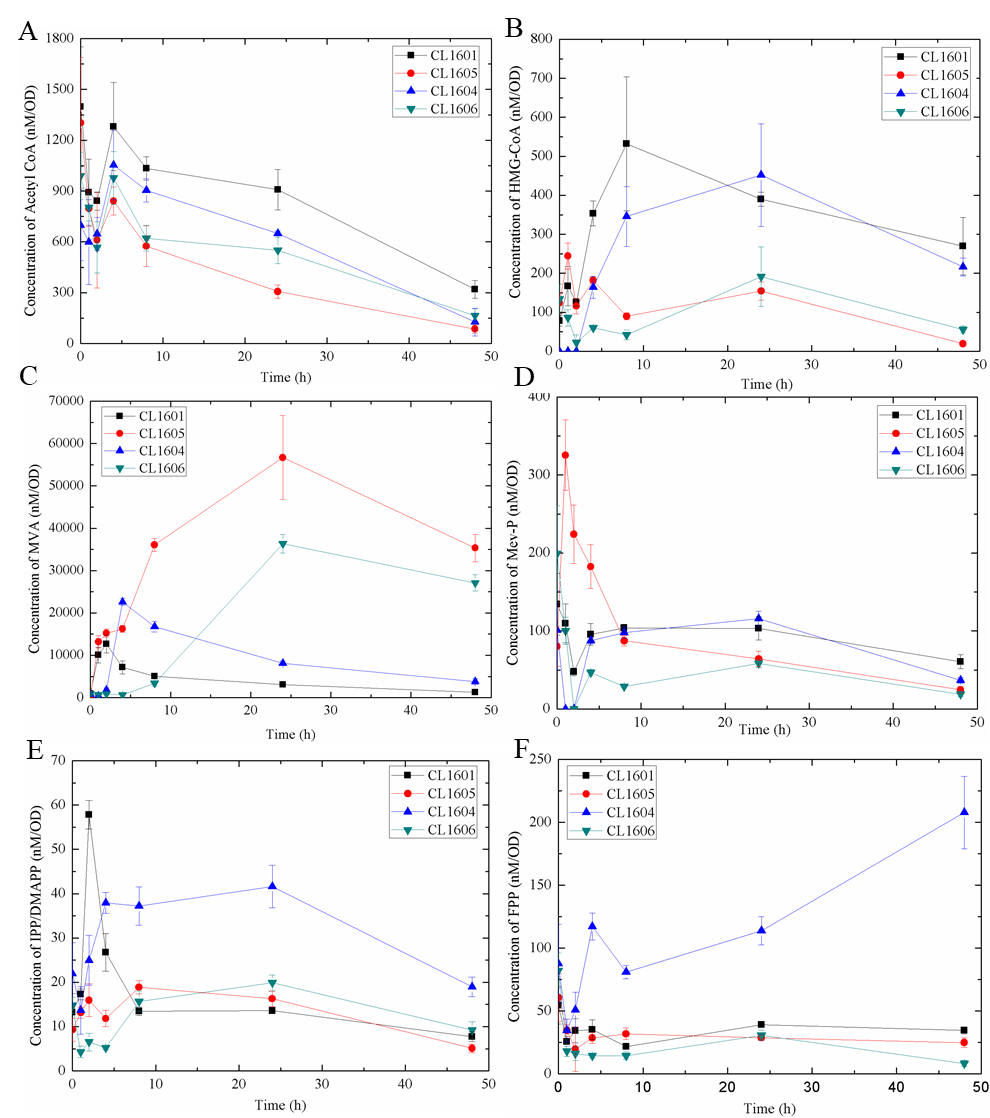


**Figure S10.** Targeted proteomic analysis of pathway enzymes for the inducible control system (strain CL1601 and CL1605), and dynamic control system (strain CL1604 and CL1606) from induction time (0 h) to 48 h. The peak areas of protein (A) AtoB, (B) HMGS, (C) HMGR, (D) MK, (E) PMK, (F) PMD, (G) Idi, (H) ispA and (I) coEIZS were also analyzed by LC-MS. Data represent averages from three replicates with error bars showing standard deviation.


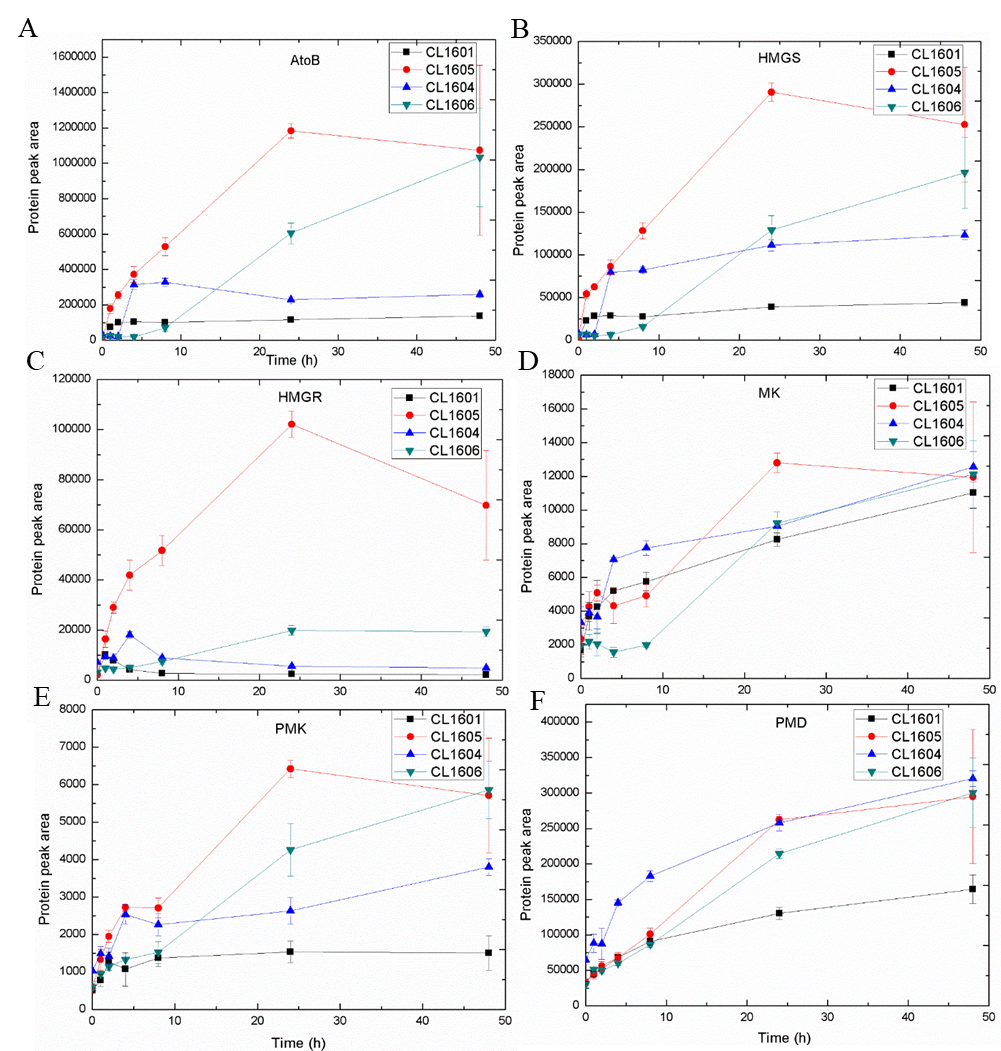


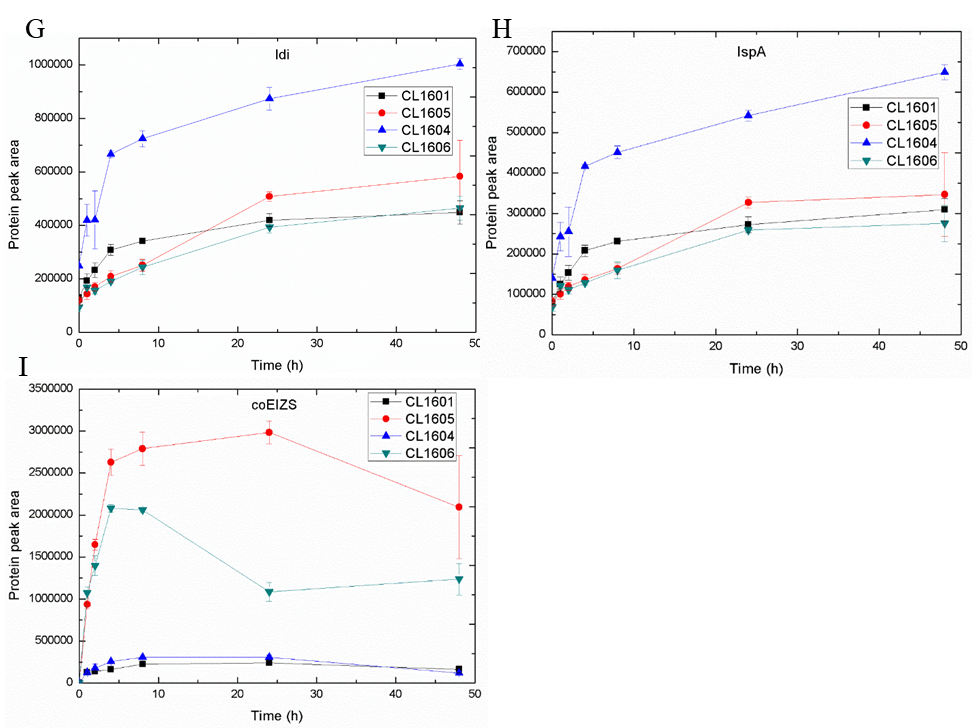


**Supplementary Methods**

**Sesquiterpene synthase expression and purification**

Sesquiterpene synthases were put on the plasmid pTrc99A with His-tag and were transformed into *E. coli* DH1 for protein expression and identification. Colonies harboring plasmids JBEI-15856, JBEI-15855, JBEI-15854 and JBEI-15853 were inoculated into LB medium containing ampicillin (100 mg/mL), and grown overnight. The seed culture was diluted in TB medium supplemented with 0.4% glycerol and 100 mg/mL ampicillin and incubated at 37°C until OD_600_ reached 0.6. The cell culture was supplemented with IPTG to final concentration of 0.5 mM and cultured at 30°C for protein expression for 6 h. Cells were collected by centrifugation and resuspended in 50 mM Tris–HCl (pH 7.5) buffer containing 300 mM NaCl and 10 mM imidazole. Cells were lysed by sonication and the supernatant and crude cell extracts were used to run SDS-PAGE.

HisTrap FF crude columns (Sigma-Aldrich, USA) were used in the protein MrTPS2 purification offering the convenience of simple, one-step purification of His-tagged proteins directly from homogenized, unclarified cell lysate by immobilized metal ion affinity chromatography (IMAC). Optimized purification of His-tagged MrTPS2 protein from homogenized, unclarified cell lysates was implemented with fast protein liquid (FPLC) chromatography system directly. Finally, the purified protein was used to run SDS-PAGE again.

**Metabolites analysis**

Colonies of strain CL1601, CL1604, CL1605 and CL1606 were picked up from the plates and inoculated into LB medium for shaking overnight at 37°C. The seed cultures were diluted into 60 mL of fresh EZ rich medium in 250 mL flasks to OD_600_ of 0.05. The culture was induced by 0.5 mM IPTG at OD_600_ ~ 0.7 and samples were collected prior to induction (0), 1, 2, 4, 8 24 and 48h after induction for metabolite analysis. The cell pellet of each sample was collected after centrifugation and was quenched by adding 250 µL of methanol on ice. 250 µL of water was added into the methanol lysate and the lysate was mixed thoroughly. The supernatant was collected into a 3,000 Da MW/CO centrifuge filter tube after centrifuging the methanol /water lysate for 3 mins, and then was filtered for 90 mins at 13,000 x g, 4°C. Finally, 500 µL water was added into the resulting filtrate, frozen in liquid nitrogen, and was lyophilized for 1-2 days.

Freeze dried pellets were resuspended in 90 µL of mobile phase and metabolites were separated via a SeQuant® ZIC®-pHILIC guard column (20-mm length, 2.1-mm internal diameter, and 5-µm particle size; from EMD Millipore, Billerica, MA, USA), in series with a short SeQuant® ZIC®-pHILIC column (50-mm length, 2.1-mm internal diameter, and 5-µm particle size) and a long SeQuant® ZIC®-pHILIC column (150-mm length, 2.1-mm internal diameter, and 5-µm particle size) using an Agilent Technologies 1200 Series Rapid Resolution HPLC system (Agilent Technologies, Santa Clara, CA, USA). Thus, the mobile phase flow path was from guard column → short column → long column → time-of-flight mass spectrometer (TOF-MS). Sample injection volume, autosampler tray temperature and column compartment were set to 2 µL, 6°C, and 40°C, respectively. The mobile phase was composed of 10 mM ammonium carbonate (Fluka Analytical, Sigma-Aldrich, St. Louis, MO, USA) and 118.4 mM ammonium hydroxide (from a 14.8 M ammonium hydroxide solution, EMD Chemicals, Billerica, MA, USA) in acetonitrile-water (60.2:39.8, v/v). Metabolites were eluted isocratically via a flow rate of 0.18 mL/min from 0 to 5.4 min, which was increased to 0.27 mL/min from 5.4 to 5.7 min, and held at this flow rate for an additional 5.4 min, giving a total run time of 11.1 min. The HPLC system was coupled to an Agilent Technologies 6210 TOF-MS system. Electrospray ionization (ESI) was conducted in the negative ion mode for the detection of [M - H]^-^ ions. Data acquisition and processing were performed by the Agilent MassHunter software package.

**Targeted proteomics**

For proteomic analysis, the methods of the sample preparation, LC-MS analysis and proteomic quantitative analysis used in this study were previously described [1, 2].

Briefly, protein lysis and precipitation were achieved by using a chloroform-methanol extraction. The cell pellets were transferred to 1.5 mL tubes, followed by the addition of 400 µL of methanol, 100 µL of chloroform, and 300 µL of water, with vortexing in between each addition. The samples were centrifuged at 21,000 x *g* for 1 minute for phase separation. The methanol and water top layer was removed, then 300 µL of methanol was added and the sample was vortexed. The samples were centrifuged at 21,000 x *g* for 2 minutes to isolate the protein pellet. The protein pellet was dried for 5 minutes at 30ºC in a vacuum concentrator and resuspended in 100 mM ammonium bicarbonate with 20% methanol. The protein concentration was measured using the DC Protein Assay Kit (Bio-Rad, Hercules, CA) and bovine serum albumin to generate the standard curve. A total of 200 µg of protein from each sample was digested with trypsin for shotgun proteomic analysis. The protein was reduced by adding tris 2-(carboxyethyl) phosphine (TCEP) at a final concentration of 5 mM and incubating at room temperature for 30 minutes. The protein was then alkylated by adding iodoacetamide at a final concentration of 10 mM and incubating for 30 minutes in the dark. Trypsin was added at a ratio of 1:50 trypsin:total protein and the samples were incubated overnight at 37ºC.

Peptides were analyzed using an Agilent 1290 liquid chromatography system coupled to an Agilent 6460 QQQ mass spectrometer (Agilent Technologies, Santa Clara, CA). The peptides (20 µg) were separated on an Ascentis Express Peptide ES-C18 column (2.7 µm particle size, 160 Å pore size, 10 cm length x 2.1 mm i.d., coupled with a 5 mm x 2.1 mm i.d. guard column; Sigma-Aldrich, St. Louis, MO) operating at a flow rate of 400 µL/min and heated to 60ºC. The chromatographic conditions were as follows: initial condition 98% Buffer A (99.9% water, 0.1% formic acid)* and 2% Buffer B (99.9% acetonitrile, 0.1% formic acid), held constant for 2 minutes, increased to 10% Buffer B in 0.5 minutes, increased to 40% Buffer B over 3.5 minutes, increased to 90% Buffer B in 0.5 minutes, held constant at 90% B for 2 minutes, then returned to 2% B in 0.5 minutes where it was held for 1 minute prior to re-equilibrate the column for the next sample. The data were analyzed by using Skyline (version 3.6) and peptide quantification was achieved by summing the integrated peak areas of the SRM transitions. Peptide abundances of the same protein were summed to assign abundance to that protein.

**Energy calculation of the sesquiterpane products**

The thermodynamic parameters of the compounds were by computationally studied, performed using Gaussian 09 software by B3LYP method at 6-311+G(2d,p) basis set level [3, 4]. The specific energy was calculated as the followed steps:

1) The isodesmic reaction:

C_15_H_26_ + 4 CH_4_= 2 C_2_H_6_ + 3 C_5_H_10_

According to the isodesmic reactions, the theoretical enthalpy of epi-isozizaane, pentalenane and α-isocomane is calculated. The total energy (E_tot_) of compounds epi-isozizaane, pentalenane, α-isocomane, methane, ethane and cyclopentane are -1540.997 MJ/mol, -1540.982 MJ/mol, -1540.946 MJ/mol, -106.287 MJ/mol, -209.433 MJ/mol, and -515.750 MJ/mol calculated by the software Gaussian 09. The enthalpy value of compounds methane, ethane and cyclopentane is from the thermodynamic database, they are -74.847 KJ/mol, -84.667 KJ/mol and -123.14 KJ/mol.

For epi-isozizaane, the standard enthalpy of formation of the isodesmic reaction is:

∆H = 2 ∆H (ethane) + 3 ∆H (cyclopentane) - ∆H (epi-isozizaane)- 4 ∆H (methane)

= 2 E_tot_ (ethane) + 3 E_tot_ (cyclopentane) - E_tot_ (epi-isozizaane)- 4 E_tot_ (methane)

Then:

-∆H (epi-isozizaane) = 2 E_tot_ (ethane) + 3 E_tot_ (cyclopentane) - E_tot_ (epi-isozizaane) - 4 E_tot_ (methane) - [2 ∆H (ethane) + 3 ∆H (cyclopentane) - 4 ∆H (methane)]

= 2× (-209.433 MJ/mol) + 3× (-515.750 MJ/mol) - (-1540.997 MJ/mol) - 4 × (-106.287 MJ/mol) - [2 × (-84.667 KJ/mol) + 3 × (-123.14 KJ/mol) - 4 × (-74.847 KJ/mol)] = -268.366 KJ/mol.

∆H (epi-isozizaane) = - 268.366 KJ/mol

2) Combustion reaction equation:

C_15_H_26_ +21.5 O_2_=15 CO_2_+13 H_2_O

According to the thermodynamic database, the enthalpy value of compounds O_2_, CO_2_ (gas) and H_2_O (gas) are 0, -393.511 KJ/mol and -241.825 KJ/mol.

The standard enthalpy of formation of the combustion reaction equation:

∆H=15∆H (CO_2_) +13×∆H (H_2_O)-∆H (epi-isozizaene) - 21.5 ∆H (O_2_)

=15× (-393.511 KJ/mol) +13× (-241.825 KJ/mol) - (-268.366 KJ/mol) - 21.5× 0

= -8788.024 KJ/mol

Specific energy (epi-isozizaane) = E_tot_ / molar mass = (8788.024 KJ/mol) / (206.367 g/mol) =42.584 MJ/kg.

Calculated by the same method, the specific energy of pantalenane and α-isocomane is 42.609 MJ/kg and 42.783 MJ/kg respectively. In this calculation method, the enthalpy of vaporization of water changing with temperature was not considered in the calculation method. The specific energy of compounds will increase 2.559 MJ/kg (using vaporization enthalpy ΔH_vap_ (100°C) = 40.63 kJ/mol to calculate) when the heat release of water from gas into liquid was included.

**References**

1. Alonso-Gutierrez J, Chan R, Batth TS, Adams PD, Keasling JD, Petzold CJ,et al. Metabolic engineering of *Escherichia coli* for limonene and perillyl alcohol production. *Metab Eng.* 2013;19:33-41.

2. Redding-Johanson AM, Batth TS, Chan R, Krupa R, Szmidt HL, Adams PD, et al. Targeted proteomics for metabolic pathway optimization: application to terpene production. *Metab Eng.* 2011;13:194-203.

3. M. J. Frisch GWT, H. B. Schlegel, G. E. Scuseria, M. A. Robb, J. R. Cheeseman, G. Scalmani, et al.. *Gaussian 09, Revision A.1.* Gaussian Inc.: Wallingford, CT; 2009.

4. Almatarneh MH, Al-Shamaileh E, Ahmad ZM, Abu-Saleh AAAA, Elayan IA. A Computational Study of the Ozonolysis of Phenanthrene. *Acta Physica Polonica A.* 2017;132:1149-1156.
